# The Role of Vitamin D in Autoimmune Diseases

**DOI:** 10.3390/ijms27010555

**Published:** 2026-01-05

**Authors:** Federica Vincenzi, Carlo Smirne, Stelvio Tonello, Pier Paolo Sainaghi

**Affiliations:** 1 Department of Translational Medicine, Università del Piemonte Orientale, 28100 Novara, Italy; federica.vincenzi@uniupo.it (F.V.); stelvio.tonello@med.uniupo.it (S.T.); pierpaolo.sainaghi@med.uniupo.it (P.P.S.); 2 Interdisciplinary Research Centre of Autoimmune Diseases (IRCAD), Università del Piemonte Orientale, 28100 Novara, Italy

**Keywords:** vitamin D, immune system, autoimmune diseases, autoimmunity, cholecalciferol, calcitriol, rheumatoid arthritis, multiple sclerosis, systemic lupus erythematosus, type 1 diabetes mellitus

## Abstract

Vitamin D is a steroid hormone whose relevant immunomodulatory role has been widely described. Therefore, its contribution to the pathogenesis of immune-mediated diseases is an important and ongoing matter of research. Specifically, the active form of vitamin D, i.e., 1,25-dihydroxyvitamin D, through the interaction with its receptor, exerts different activities on the innate and adaptive immune system, among which are suppression of inflammation and promotion of tolerogenic responses. Indeed, vitamin D insufficiency/deficiency has been related to the pathogenesis and/or disease activity of several autoimmune diseases, including, amongst others, multiple sclerosis, rheumatoid arthritis, systemic lupus erythematosus, and type 1 diabetes mellitus. Based on these premises, in this review, we will describe the main molecular mechanisms modulated by vitamin D in the regulation of immune responses, including the induction of immune tolerance. Moreover, we will focus on the current knowledge regarding the contribution of vitamin D depletion to the aforementioned autoimmune diseases, seeking to provide evidence as to why its supplementation in the context of these immune-mediated disorders may potentially ameliorate disease activity and its related clinical manifestations.

## 1. Introduction

Vitamin D is a fat-soluble vitamin mainly involved in bone remodeling, through the regulation of calcium reabsorption from bone and intestine. Due to its structure, vitamin D acts as a hormone, influencing cellular differentiation and proliferation, immune system regulation, neural function, and cardiovascular health [1]. Despite its classical function, increasing evidence has been gathered on the immunomodulatory effects of vitamin D on both innate and adaptive immune systems. This knowledge arises from studies performed in 1903 by Dr. Nils Filsen, who cured the epidermal form of tuberculosis (lupus vulgaris) using light irradiation. The following findings regarding the synthesis of vitamin D in the skin after ultraviolet (UV) light exposure opened a window to the possible immunomodulatory role of vitamin D, particularly in the treatment of mycobacterial infections, such as tuberculosis and leprosy [2]. Further studies showed that both vitamin D receptor (VDR) and metabolizing enzymes are expressed in a plethora of immune cells, including dendritic cells, lymphocytes, monocytes, and macrophages. These findings highlight the capacity of immune cells to synthetize vitamin D, producing both VDR-mediated paracrine or intracrine responses [2]. In light of the current knowledge about vitamin D’s immunomodulatory role, researchers focused on the possible contribution of vitamin D in autoimmune diseases and immune-related disorders. Indeed, the association between vitamin D deficiency and an increased risk of autoimmune diseases has been described [3]. Several immune-mediated diseases have been the topic of research, including rheumatoid arthritis (RA), systemic lupus erythematosus (SLE), multiple sclerosis (MS), and type 1 diabetes mellitus (T1D), which will be the focus of this review. However, it is essential to give a brief overview of the relationship between vitamin D and less studied diseases including psoriasis, inflammatory bowel disease (IBD), autoimmune thyroiditis, and celiac disease.

Low levels of vitamin D have been reported in patients with psoriasis, a known chronic inflammatory autoimmune disease affecting the skin [4]. Moreover, due to their anti-inflammatory properties, vitamin D and derivatives seem to be effective adjuvant treatments for psoriasis, both orally and topically administered [5]. Another immune-mediated condition is IBD, characterized by chronic intestinal inflammation. This persistent inflammation leads to malabsorption, which can contribute to increased risk of vitamin D deficiency. Moreover, vitamin D/VDR signaling can modulate intestinal epithelium permeability, the release of antimicrobial peptides, and mucus secretion. Therefore, its supplementation may help restore mucosal integrity and reduce inflammation [6]. Celiac disease is another autoimmune intestinal disorder triggered by gluten intake, resulting in chronic inflammation and villous atrophy [7]. In celiac disease, intestinal barrier integrity is altered, and, as for IBD, vitamin D deficiency may be caused by malabsorption. Even if vitamin D may help reduce inflammation, no conclusive results have been gathered regarding the effects of its supplementation in the context of celiac disease [8]. An association between celiac disease and autoimmune thyroiditis, including Hashimoto’s thyroiditis and Graves’ disease, has been reported. This could be due to the diversity of the gut microbiome and nutrient deficiency, including vitamin D, which plays an important role in regulating gut, thyroid, and immune system function [7]. Interestingly, vitamin D dampens excessive inflammation and T cell activation; therefore, it may contribute to reducing tissue damage in autoimmune thyroiditis [9].

In our review, we will explore the mechanisms by which vitamin D regulates both the innate and adaptive immune systems and the relationship between vitamin D and immune-related disorders, focusing on RA, MS, SLE, and T1D.

## 2. Vitamin D Metabolism and Function

**Figure 1:**
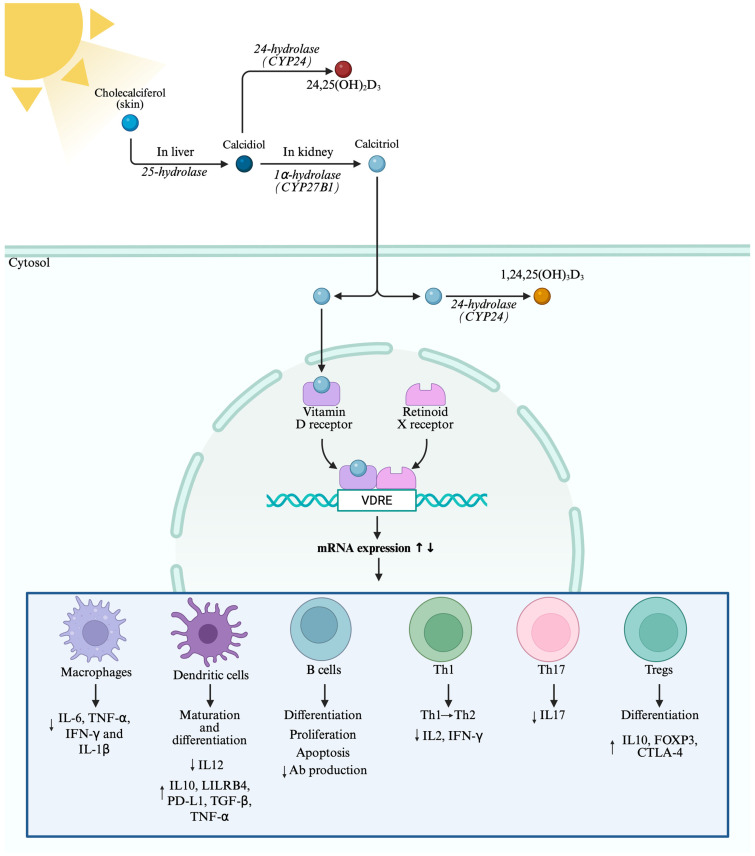
Effects of calcitriol on the immune system. Cholecalciferol is produced in the skin after exposure to UV-B light and is converted to calcidiol in the liver by 25-hydroxylase and later in the kidney to calcitriol by 1α-hydroxylase (CYP27B1). To avoid toxic accumulation, calcidiol and calcitriol can be further hydroxylated by 24-hydroxylase to 24,25-dihydroxycholecalciferol (24,25(OH)_2_D_3_) and 1,24,25-trihydroxycholecalciferol (1,24,25(OH)_3_D_3_), respectively. In the cytosol, calcitriol is recognized by the VDR-RXR complex, which translocates to the nucleus and binds to VDREs, regulating the expression of several genes involved in the innate and adaptive immune responses. In macrophages, calcitriol can reduce the production of proinflammatory cytokines, including interleukin (IL)-6, tumor necrosis factor (TNF)-α, interferon (IFN)-γ, and IL-1β. Calcitriol can also modulate maturation and differentiation of dendritic cells, inducing a tolerogenic status characterized by the reduction in cluster differentiation (CD)40, CD86, CD1a, and major histocompatibility complex (MHC)-II expression. In addition, calcitriol reduces IL-12 and increases IL-10, leukocyte immunoglobulin-like receptor subfamily B member 4 (LILRB4), Programmed Cell Death 1 Ligand (PD-L1), transforming growth factor β (TGF-β), and TNF-α production by dendritic cells. Regarding adaptive immune cells, calcitriol can induce the switch from T helper (Th)1 proinflammatory cells to Th2 anti-inflammatory cells, also reducing IL-2 and IFN-γ production by Th1 cells and IL-17 by Th17 cells. The anti-inflammatory effect of calcitriol is also underlined by the induction of regulatory T cells (Tregs) differentiation, characterized by increased expression of IL-10, forkhead box P3 (FOXP3), and Cytotoxic T-Lymphocyte Antigen 4 (CTLA-4). The relationship between calcitriol and B cells has not been widely investigated. However, it is known that calcitriol modulates differentiation, proliferation, and apoptosis of B cells and reduces antibody production. Created in BioRender. Minisini, R. (2026) https://BioRender.com/gsw8cts, accessed on 22 December 2025.

Despite its name, vitamin D is a hormone precursor which can be endogenously synthetized after UV-B exposure or can be introduced with the diet. Vitamin D encompasses vitamin D_2_ (ergocalciferol), derived from plants, and vitamin D_3_ (cholecalciferol), which is synthetized in animals [10]. Upon UV-B exposure, the 7-dehydrocholesterol in the skin is photoconverted into cholecalciferol, which binds to the Vitamin D Binding Protein (VDBP), and it is transported to the liver. Cholecalciferol is the biologically inactive form of vitamin D. Therefore, it is subjected to a series of modifications to convert it to calcitriol, also known as 1,25-dihydroxycholecalciferol, the active form of vitamin D, as shown in Figure 1. In the liver, a plethora of enzymes belonging to the cytochrome P450 (CYP) class, including CYP27A1 and CYP2R1, hydroxylate cholecalciferol in position C25, producing calcidiol, also known as 25-hydroxycholecalciferol [11]. This metabolite is the most abundant form in the bloodstream, and, due to its long half-life, it is the best indicator of vitamin D status. Indicatively, serum levels of calcidiol should be greater than 30 ng/mL (75 nmol/L) but not exceeding 150 ng/mL (374 nmol/L). Moreover, levels between 21–29 ng/mL (52.5–72.5 nmol/L) indicate vitamin D insufficiency, while deficiency can be suggested with calcidiol levels lower than 20 ng/mL (50 nmol/L) [12,13]. However, no definitive consensus has been reached regarding the normal circulating levels of vitamin D.

Calcidiol is further converted in the proximal tubule of the kidney through the enzyme 1α-hydroxylase (CYP27B1) to calcitriol, the hormonally active form. Studies also reported the presence of CYP27B1 in extrarenal sites, including placenta, bone, and various immune cells, underlying their capacity to produce calcitriol for intracrine and paracrine effects [14]. Calcitriol is then transported using VDBP in the target organs, including kidneys, gut, and bone, where it can exert its genomic and non-genomic functions. Genomic functions are mediated by the binding of calcitriol to cytosolic VDR in the target cell. This interaction induces VDR phosphorylation and heterodimerization with retinoid-X-receptor (RXR). The calcitriol–VDR-RXR complex translocates into the nucleus and binds to vitamin D response elements (VDREs), inducing either activation or repression of genes regulating different biological activities. This process is also mediated by the interaction of VDR with transcriptional coactivators or corepressors [15,16]. Through VDR, calcitriol can activate a negative feedback loop, promoting the expression of the gene encoding for CYP24. This enzyme has 24-hydroxylase activity, responsible for the hydroxylation in position C24 of both calcidiol and calcitriol, producing inactive metabolites and preventing toxic accumulation [17]. Moreover, calcitriol can downregulate the expression of both the CYP27B1 gene in the kidney and of the parathyroid hormone (PTH) gene. This negative feedback decreases the synthesis of both calcitriol and PTH, which modulates calcium homeostasis and positively regulates CYP27B1 [18,19]. In addition to its genomic effects, calcitriol can also bind to membrane VDRs, activating a series of non-genomic pathways, including calcium and mitogen-activated protein kinase (MAPK) signaling [15]. These effects are mediated by VDR’s interaction with other molecules, including Inhibitor Of Nuclear Factor Kappa B Kinase Subunit Beta (IKKβ), a regulator of the nuclear factor kappa B (NF-κB) pathway, signal transducer and activator of transcription (STAT)1, c-Jun, and β-catenin. Through these pathways, calcitriol can modulate the innate and adaptive immune system and antiviral response as well as cell survival [20]. Currently, it is known that VDR is widely expressed in several tissues, including prostate, breast, brain, colon, and immune cells. Referring to the latter, VDR has been found to be highly expressed in T lymphocytes, dendritic cells, monocytes, and macrophages, underlying the variety of physiological actions potentially exerted by this molecule [21].

In the following sections, we will describe the effects of vitamin D on innate and adaptive immunity. It is essential to set out that in the existing literature, the term “vitamin D” is frequently used without explicit specification of the molecular form administered. For clarity and consistency, in this review, the term “vitamin D” will be used to denote studies in which the specific vitamin D metabolite or analogue is not explicitly stated, unless otherwise indicated.

## 3. Vitamin D and Innate Immunity

The innate immune system serves as the body’s first line of defense, mounting a non-specific response against invading pathogens. It is characterized by complement activation and antibacterial response as well as antigen presentation to adaptive immune cells. In this context, several studies have highlighted the involvement of vitamin D in the regulation of the innate immune system, depicting its possible contribution to pathogen clearance as well as to the interplay between innate and adaptive immunity [22]. Figure 1 summarizes the effects of vitamin D in both innate and adaptive immune responses.

### 3.1. Macrophages

Monocytes and macrophages, key players in the innate immune system, are also targets of vitamin D, which modulates their antimicrobial and anti-inflammatory responses. These cells can recognize pathogen-associated molecular patterns (PAMPs) on the surface of pathogens, producing cytokines and exerting a phagocytic activity, contributing to the resolution of the infection through the interaction of PAMPs with toll-like receptors (TLRs) on monocytes and macrophages [23]. Upon TLR activation, a cascade of intracellular events leads to the synthesis of the active vitamin D metabolite, calcitriol, within macrophages. Indeed, studies performed on human macrophages treated with *Mycobacterium tuberculosis* have shown that TLR, mainly TLR1 and TLR2, interaction with PAMPs induces the activation of CYP27B1 and VDR, suggesting an intracrine synthesis of calcitriol and the subsequent signaling activated by the binding to VDR [24]. Therefore, particularly in granulomatous disorders such as tuberculosis, sarcoidosis, and some lymphomas, the excessive production of calcitriol by macrophages stimulates intestinal calcium absorption, leading to hypercalcemia and hypercalciuria [25]. This effect is exacerbated by the presence in macrophages of a non-functional variant of 24-hydroxylase, which prevents the negative regulation of vitamin D production [26].

Conversely to renal enzymes, which are regulated by PTH, the activation of vitamin D-metabolizing enzymes in extrarenal sites is mediated by the circulating levels of calcidiol, the most abundant form in the bloodstream. Indeed, CYP27B1 in monocyte/macrophages is regulated by both calcidiol and different types of cytokines, such as interferon (IFN)-γ, interleukin (IL)-1, and tumor necrosis factor (TNF)-α [26]. Experiments performed on peripheral blood mononuclear cells (PBMCs) of healthy individuals have shown that IFN-γ and lipopolysaccharide (LPS) stimulation enhance calcitriol synthesis, demonstrating that in vitro differentiation of immature monocytes in macrophages is associated with increased production of calcitriol [27]. Reinforcing these findings, Stoffels et al. established that the promoter of the CYP27B1 gene harbors binding sites for inflammation-responsive transcription factors, evidencing the contribution of inflammatory pathways to the enzyme’s regulation [28]. Furthermore, they established that the activity of 1α-hydroxylase is blocked by inhibitors of MAPK, Janus kinase (JAK), and NF-κB, suggesting the contribution of these pathways in the regulation of CYP27B1 activity and therefore in calcitriol synthesis [28].

Beyond its differentiating properties, vitamin D exerts potent anti-inflammatory effects, primarily by modulating NF-κB signaling and MAPK pathways. Studies on murine RAW 264.7 cells reported that calcitriol attenuates LPS-induced NF-κB gene enhancer B cells inhibitor alpha (IκBα) phosphorylation, leading to the inhibition of NF-κB. This results in the transcriptional downregulation of microRNA (miR)-155, a critical modulator of innate immunity, which in turn increases suppressor of cytokine signaling (SOCS) translation, thereby inhibiting LPS-mediated production of pro-inflammatory cytokines such as IL-6, TNF-α, IFN-γ, and IL-1β [29]. These findings have been confirmed by Cohen-Lahav et al., who reported that calcitriol can also increase the levels of IκBα, thus reducing the production of pro-inflammatory stimuli [30]. The synthesis of inflammatory cytokines following LPS stimulation is also mediated by the members of the MAPK family, comprising extracellular-signal-regulated kinase (ERK), c-Jun N-terminal kinase (JNK), and p38. In this regard, in vitro studies showed that pretreatment of human PBMCs with physiological concentrations of calcidiol, between 30–50 ng/mL, and of calcitriol, between 1–10 nM, inhibit LPS-induced p38 activation. Inhibition of p38 significantly decreases both IL-6 and TNF-α mRNA expression [31].

### 3.2. Dendritic Cell Modulation

Dendritic cells (DCs) are antigen-presenting cells responsible for the regulation of both innate and adaptive immune response. DCs are an heterogeneous population that can be divided into myeloid-derived (mDCs) and lymphoid-derived DCs (plasmacytoid, pDCs) according to the expression of cluster differentiation (CD)11c. mDCs share several features with monocytes, including the expression of CD33, CD13, and CD11c, along with low levels of CD123, and they are major producers of IL-12. In contrast, pDCs resemble plasma cells, characterized by high expression of CD4, CD62L, and CD123, and they mainly produce IFN-α.

Upon antigen exposure, mature DCs increase their antigen processing ability and display major histocompatibility complex (MHC)-I and MHC-II on their surface for antigen presentation as well as costimulatory molecules for T cell activation, including CD80 and CD86 [32,33]. Studies have identified similarity between DCs and macrophages in the expression pattern of CYP27B1 and VDR. However, mature DCs present elevated levels of CYP27B1 and reduced levels of VDR, while immature DCs highly express VDR. According to these findings, it can be suggested that calcitriol produced by mature DCs does not exert an intracrine effect but acts on immature DCs, allowing some DCs to mature but preventing an exaggerated response and possible pathological manifestations [34].

Indeed, it has been reported that calcitriol interferes with DC differentiation and maturation, in particular acting on mDCs, inducing a tolerogenic state (tDCs). This phenotype is characterized by expression of CD11c and downregulation of CD40, CD80, CD86, CD1a, and MHC-II [35]. An in vitro study of Penna et al. on human-derived DCs demonstrated that calcitriol, beyond inhibiting differentiation and maturation of immunogenic DCs, is very effective in reducing the secretion of IL-12, enhancing IL-10 production, and promoting DC apoptosis, thus resulting in T cell hyporesponsiveness [36]. Moreover, calcitriol upregulates the expression of T-cell-inhibitory molecules on tDCs, including leukocyte immunoglobulin-like receptor subfamily B member 4 (LILRB4) and Programmed Cell Death 1 Ligand (PD-L1), induces IL-10, transforming growth factor β (TGF-β), and TNF-α production, and downregulates NF-κB expression. These mechanisms result in decreased T cell activation and a shift in T cell polarization from a T helper (Th)1 and Th17 response to a regulatory T cells (Tregs) response [33]. In line with these affirmations, vitamin D may indirectly regulate the adaptive immune system, particularly T cell differentiation, through the modulation of DC differentiation, maturation, and apoptosis.

## 4. Vitamin D and Adaptive Immunity

Besides the indirect regulation of T cells mediated by DCs, the presence of VDR and CYP27B1 in B and T lymphocytes suggests that vitamin D can directly modulate these cells. Moreover, studies have reported that B and T cell activation leads to an increase in the expression of both VDR and CYP27B1. However, unlike the effects on innate immunity, vitamin D generally suppresses adaptive immunity [37].

### 4.1. B Cells

Only a limited number of studies have investigated the relationship between vitamin D and B cells. However, the expression of VDR- and vitamin D-metabolizing enzymes in B cells, like in other immune cells, has been reported [38]. Studies have shown that calcitriol modulates B cell production of IL-10. Heine et al. describe that the calcitriol–VDR interaction with VDREs in the promoter of IL-10 upregulates its expression in human CD40/IL-4-activated B cells [39]. Evidence suggests that vitamin D regulates B cell processes, including differentiation, proliferation, and apoptosis, and suppresses antibody production [40,41]. These effects may be relevant in the pathogenesis of inflammatory and autoimmune diseases.

### 4.2. T Cells

T cells can be subdivided into distinct functional subsets. CD4^+^ Th cells support B cell antibody (Ab) production and other cells in pathogen killing. CD4^+^ Th cells can differentiate into Th1 cells, mediators of response to viruses and intracellular infections, Th2 cells, which secrete IL-4, IL-5, and IL-13 and contribute to response to parasitic infections, B cell activation, and Ab production. Th17 cells act on extracellular pathogens and produce IL-17, Granulocyte–Macrophage Colony-Stimulating Factor (GM-CSF), and IL-22. Moreover, the presence of Th9, Th22, and T follicular helper cells has been reported. Another subset of T cells comprises CD8^+^ cytotoxic T cells, which are responsible for apoptosis of viral- or bacterial-infected cells. Persistent T cell activation and failure of regulatory control can then cause immune-mediated diseases. To avoid this uncontrolled T cell activation, Tregs, which are primary mediators of peripheral tolerance, can, in turn, exert a protective role by suppressing T cell responses [42].

Vitamin D reduces the production of pro-inflammatory cytokines by Th1 and Th17 cells, including IFN-γ, IL-2, and IL-17, while promoting the induction of IL-10-producing Tregs expressing forkhead box P3 (FOXP3) and Cytotoxic T-Lymphocyte Antigen 4 (CTLA-4) [43]. Vitamin D can modulate intracellular pathways mediating IL-2 production, leading to reduced synthesis. Specifically, calcitriol suppresses Nuclear Factor Of Activated T Cells (NFAT)/Activator Protein-1 (AP-1) protein complex formation, implicated in IL-2 expression. The subsequent association of the ligand–VDR-RXR complex to the NFAT binding site on IL-2 promoter inhibits its expression. The reduced IL-2 production prevents activation and proliferation of T cells [44]. Moreover, by inhibiting IFN-γ, vitamin D reduces antigen presentation and T cell recruitment. Th1 cells play a major role in graft rejection and autoimmune diseases, while Th2 cells act as regulatory cells. The inhibition of IL-2 and IFN-γ synthesis mediated by calcitriol induces a switch from a Th1 to Th2 phenotype, thus reducing the risk of autoimmunity and graft rejection [44,45].

However, the modulatory effects of the hormonally active form calcitriol on Th2 differentiation and cytokine production have yielded contradictory findings. Indeed, some studies reported that calcitriol upregulates GATA binding protein 3 (GATA-3) and c-Maf expression, two Th2-specific transcription factors, therefore promoting IL-4 and IL-5 secretion [35,46]. On the contrary, Biswas et al. reported that calcitriol stimulation of murine Th2 cells led to decreased IL-4 and IL-13 levels, accompanied by increased production of IL-10 [47]. Moreover, they observed decreased expression of GATA-3, Growth Factor Independent 1 Transcriptional Repressor (Gfi1), and Interferon Regulatory Factor 4 (IRF4) in calcitriol-treated Th2 cells [47]. Additionally, calcitriol suppresses IFN-γ production in Th1-polarized murine cells and inhibits IL4 in Th2-polarized murine cells; however, it promotes IL-4 production in non-polarizing conditions [48].

Moreover, additional studies reported that calcitriol can regulate a wide range of genes involved in CD4^+^ T cell proliferation and apoptosis. In particular, calcitriol can upregulate the expressions of the genes encoding for CTLA-4, CD38, CYP24A1, the cytokines IL-10 and IL-6, and many transcription factors, including c-Jun, BTB Domain And CNC Homolog 1 (BACH), and STAT3. Interestingly, type 1 (IFN-γ) and type 17 (IL-17, IL-22, and IL-26) cytokine expression in Th cells is suppressed by calcitriol [49]. Given the effects of vitamin D on both innate and adaptive immunity, supplementation may improve immune function and modulate immune-mediated diseases. In the following sections, we will focus our attention on the current knowledge about vitamin D in autoimmune diseases.

## 5. Vitamin D in Autoimmune Diseases

Autoimmune diseases, including RA, MS, SLE, and T1D, are characterized by immune dysregulation. In particular, aberrant T cell reactivity and autoantibody production contribute to tissue damage. Vitamin D immunomodulatory effects have been a topic of research in the context of autoimmunity. In this setting, vitamin D deficiency has been reported in a range of autoimmune diseases and may impair immune tolerance, potentially contributing to disease onset [50,51]. Hence, the VITAL trial (NCT01169259) reported that 5-year supplementation with 2000 international units (IU; 1 μg vitamin D corresponds to 40 IU) [52] of vitamin D daily, with or without omega 3 fatty acids, reduces the incidence of autoimmune diseases [53]. In addition to the immunomodulatory role of vitamin D, emerging evidence has been gathered regarding the role of VDR single-nucleotide polymorphisms (SNPs) in the pathogenesis of immune-mediated disorders [54]. VDR polymorphisms may alter VDR function and signaling, thereby affecting the immunomodulatory role of vitamin D in the context of autoimmune diseases. The most relevant SNPs studied include BsmI (rs1544410, C>T), ApaI (rs7975232, A>C), TaqI (rs731236, T>C), and FokI + 30,920 (rs2228570, C>T), affecting VDR translation and mRNA stability. Moreover, studies report that the association between SNPs and several autoimmune diseases varies according to the ethnicity of the population [55]. TaqI polymorphism is characterized by a mutation in the transcription region of the VDR gene, which may result in post-transcriptional regulation alterations [56]. ApaI and BsmI polymorphisms are within intron 8, and they may affect VDR function and signaling through alternative splicing. FokI polymorphism is caused by a C-to-T transition in the start codon; therefore, it alters the structure of the final protein [57]. A TaqI heterozygous (CT vs. TT) model has been associated with MS risk in European and Asian populations [58], while the homozygote model (TT vs. CC) has been negatively associated with MS susceptibility in the Iranian population [59]. Additionally, despite no pooled association being described between TaqI SNP and RA risk, a protective role of TaqI has been reported in the African population [55]. TaqI has also been associated with reduced risk of autoimmune thyroid disease [60].

Regarding ApaI, a protective role against MS of ApaI recessive and homozygous model has been reported in Asian patients [58]. Interestingly, the ApaI homozygous model has been associated with increased risk of SLE and RA in the Italian population [61].

A BsmI heterozygous model has been reported to be associated with increased RA risk [62]. Moreover, an association between FokI polymorphism and increased RA risk in Europeans has been reported [63], while the homozygous model has been associated with decreased SLE risk in African and Asian populations [64]. As previously stated, these SNPs may alter VDR function and, therefore, the immunomodulatory role that can be exerted by vitamin D.

In the following sections, we will describe the immunomodulatory effects of vitamin D in the context of immune-mediated diseases, focusing on MS, RA, SLE, and T1D.

### 5.1. Multiple Sclerosis

MS is a progressive central nervous system (CNS) autoimmune disorder characterized by axonal demyelination, driven by self-reactive T and B cells activated in secondary lymphoid organs. After crossing the blood–brain barrier (BBB), these cells infiltrate the CNS, recognize self-antigens, and trigger an inflammatory response that leads to demyelination [65]. Several studies indicate that vitamin D deficiency is associated with MS progression, relapse, and severity, highlighting its contribution to disease pathogenesis [66,67]. Vitamin D effects in MS are attributed both to its immunomodulatory properties and its direct influence on CNS functions. Vitamin D functions as a neurosteroid, contributing to neuroplasticity and modulating immune activity within the brain [68]. Studies have shown accumulation of vitamin D in the nuclei of neurons and VDR expression in oligodendrocyte-like cells, microglia, and astrocytes, suggesting its role in the transcriptional activation of neuronal messengers. Microglia are immune cells within the CNS whose activation impacts neuron survival and has been linked with MS, schizophrenia, and other neurological disorders. In this framework, vitamin D confers neuroprotection by downregulating proinflammatory cytokines and limiting free radical release from microglia, thereby attenuating the immune response [69].

Indeed, Shirazi et al. reported that calcitriol, the active form of vitamin D, can reduce inflammation in experimental autoimmune encephalomyelitis (EAE), an animal model of MS [70]. In particular, calcitriol administration to EAE mice reduced the number of infiltrating CD11b^+^ and CD4^+^ cells as well as proinflammatory cytokine production, switching to an anti-inflammatory milieu characterized by secretion of IL-10 and IL-4. Furthermore, this study reported reduced CNS demyelination and amelioration of clinical symptoms [70]. Calcitriol supplementation also decreases the number of Th1- and Th17-infiltrating cells, key players of MS pathogenesis. Additionally, preservation of BBB permeability and integrity was observed in both the brain and spinal cord of EAE mice supplemented with calcitriol, potentially contributing to its protective effects against immune infiltration [71].

In light of the experimental evidence regarding the immunomodulatory effects of vitamin D in MS, several clinical trials evaluating the therapeutic benefit of vitamin D supplementation have been performed, as reported in Table 1.

The VIDAMS trial did not report a difference in the relapse rate between patients supplemented with low-dose (600 IU) or high-dose (5000 IU) vitamin D as an add-on to glatiramer acetate over 96 weeks [72]. In the EVIDIMIS trial, patients with relapsing-remitting MS (RRMS) or clinically isolated syndrome (CIS) were supplemented with 20,400 IU (high dose) or 400 IU (low dose) cholecalciferol, the inactive form of vitamin D, every other day for 18 months in addition to IFN β-1b. The primary endpoint consisted of the number of new T2-weighted (T2w) lesions on MRI, while among the secondary endpoints, relapse rate, disability progression, T2w lesion volume. and brain atrophy were evaluated. The low number of participants in this study did not allow for the detection of significant differences in the endpoint considered between the high- or low-dose supplementation [73,74]

However, promising results were reported in the D-Lay-MS randomized placebo-controlled trial, in which 303 untreated patients with CIS suggesting MS were randomized to the placebo group or monotherapy cholecalciferol group (100,000 IU) every 2 weeks for 24 months. Primary endpoints included the presence of disease activity, defined by MRI activity or occurrence of relapse, while secondary outcomes involve the presence of new or enlarged lesions, as well as Expanded Disability Status Scale (EDSS) and Fatigue Scale for Motor and Cognitive Functions (FSMC) scores until disease activity or at the end of follow-up. The results showed that cholecalciferol monotherapy reduces disease activity as well as the occurrence of new or enlarged lesions. However, in the two groups in the study, no significant differences in relapse and clinical scores were observed [75]. Despite no effects on the relapse rate being noticed, these results highlight the potential of high-dose vitamin D as a monotherapy in reducing disease activity and the necessity to study its implication as a supplementation to standard therapy. In this regard, in the CHOLINE study, patients with relapsing MS were randomized into either placebo or 100,000 IU cholecalciferol groups twice monthly for 96 months as an add-on to IFN β-1a (Rebif ^®^) treatment. This study evidenced a significant reduction in annualized relapse rate (ARR), T1 lesions, and EDSS score in patients who completed the study [76]. The protective effects of high-dose cholecalciferol supplementation to RRMS patients treated with IFN β-1a were confirmed by the SOLAR trial, which suggested potential benefits of vitamin D_3_ supplementation on MRI activity [77,78]. The SOLARIUM sub-study [79], enrolling 56 patients from the Netherlands cohort of the SOLAR study, investigated the effects of vitamin D supplementation on regulatory T lymphocytes as well as on cytokine production from Th cells and PBMCs. This study did not find any effect of vitamin D supplementation on Treg absolute count; however, they found differences in the proportion of anti-inflammatory IL-4^+^ Th cells as well as pro-inflammatory cytokines produced by PBMCs between treatment groups.

Despite the importance of vitamin D in regulating the immune system and the ongoing research regarding supplementation, no significant clinical benefits have been reported regarding MS progression. Further studies, enrolling an adequate number of participants, should be performed to evaluate the real contribution of vitamin D supplementation in MS pathogenesis.

### 5.2. Rheumatoid Arthritis

RA is a chronic inflammatory autoimmune disorder. RA pathogenesis is complex, but it is well known that the interplay of innate and adaptive immune cells, along with autoantibody production, such as rheumatoid factor (RF) and anti-citrullinated protein antibodies (ACPAs), drives synovial inflammation and joint destruction in RA [80].

Many immune cells contribute to RA pathogenesis, including monocytes/macrophages, which play a pivotal role by releasing inflammatory cytokines and reactive oxygen species, thus exacerbating inflammation [81,82]. Given its multiple effects on the immune system, vitamin D may influence disease course. Neve et al. reported that calcitriol-treated macrophages derived from peripheral blood of RA patients presented lower levels of both membrane and soluble TNF-α, a coordinator of the inflammatory response and a major therapeutic target for RA treatment [83]. Moreover, Zwerina et al. found increased macrophage infiltration, proinflammatory cytokine production, and osteoclastogenesis in synovia of VDR knockout human tumor necrosis factor α transgenic (VDR−/− hTNFtg) mice as compared to hTNFtg mice, suggesting a role of VDR, and therefore vitamin D, in mediating synovial inflammation and bone resorption [84].

Within the innate immune players in RA, DCs contribute to the presentation of autoantigens to B cells, promoting autoantibody production and immune complex formation. Additionally, DCs participate in the co-stimulatory activation of CD4^+^ T cells [82]. Th1 cells release TNF-α and INF-γ, contributing to initiation and sustainment of inflammation in RA, while Th17 cells, through IL-17 secretion, intensify the release of inflammatory cytokines and chemokines, thus recruiting other immune cells and contributing to cartilage degradation and bone erosion [85,86]. It has been reported that T cells in synovial fluid are relatively insensitive to calcitriol; therefore, its direct immunomodulatory effects on the tissue may be impaired. In this context, evaluation of vitamin D’s effects on peripheral cells may not provide the whole picture of the local effects of vitamin D in RA [87].

Additionally, in RA, an imbalance between Th17/Treg cells contributes to disease pathogenesis. Surely, Tregs-mediated suppressive mechanisms are essential to control autoreactivity and maintain self-tolerance. However, studies identified reduced frequency and decreased immunosuppressive activity of Tregs in RA patients’ peripheral blood [88]. It has been evidenced that vitamin D promotes IL-10^+^ Tregs differentiation and inhibits CD4^+^ T cell differentiation, which, in the context of RA, may help to restore the Th17/Treg ratio, contributing to amelioration of the disease [89]. In a separate study, T cells isolated from PBMCs of early RA patients and activated with anti-CD3/-CD28 were treated with calcitriol, with or without methotrexate (MTX). After 72 h, the proportion of Th1 and Th17 cells as well as the Th17/Treg ratio decreased in both calcitriol- and calcitriol/MTX-treated groups compared to untreated controls. However, no significant reduction was observed in the calcitriol-treated cells compared to MTX alone [90].

In a randomized, non-blinded interventional study (NCT04472481), 40 patients with active RA were randomized to receive either MTX plus hydroxychloroquine or MTX and hydroxychloroquine plus ergocalciferol (1.25 mg weekly) for 3 months. Tregs were lower in RA patients than in healthy controls at baseline. After three months of treatment, a significant elevation in Tregs in RA patients was reported, particularly in the group receiving vitamin D supplementation [91].

Vitamin D deficiency has been inversely correlated with RA prevalence and disease activity [92]. In particular, RA patients with high disease activity, compared to moderate and low, presented significantly lower serum levels of calcitriol, the hormonally active form of vitamin D [93]. A meta-analysis of 24 eligible studies performed between 1991 and 2015 involving a total of 3489 patients revealed lower levels of vitamin D in RA patients compared to healthy controls. Moreover, they evidenced a correlation between higher vitamin D levels and reduced symptoms, defined by lower disease activity and C-reactive protein (CRP) levels [94]. Additionally, our group reported increased PTH levels, irrespective of vitamin D levels, in patients with autoimmune rheumatic diseases, including RA, compared to controls, suggesting altered vitamin D metabolism [95]. Interestingly, normalization of vitamin D and PTH levels was achieved only after high-dose cholecalciferol supplementation (single 300,000 IU dose, followed by 800–1000 IU daily) for 6 months [96].

Many experimental data suggest a link between vitamin D levels and RA; however, studies on the effects of vitamin D supplementation in RA patients have not given clinically relevant results. Therefore, further studies, both in vitro and in vivo, should be performed in order to elucidate the possible clinical efficacy of vitamin D in the context of RA.

### 5.3. Systemic Lupus Erythematosus

SLE is a systemic, chronic autoimmune disease, with a prevalence ratio of 9:1 in favor of women. The etiology of SLE is not fully understood; however, it is known that genetic and environmental factors contribute to disease pathogenesis, activating the immune response and inducing B cell autoreactivity, characterized by the production of autoantibodies against nuclear and cytoplasmic antigens, such as anti-double-strand DNA antibodies (anti-dsDNA). Clinically, SLE manifests with different symptoms, which range from mild to severe. The disease commonly presents itself with cutaneous and renal manifestations (lupus nephritis, LN), but it can extend to other systems [97]. A key cutaneous manifestation of SLE is photosensitivity, which, in combination with reduced sunlight exposure and the use of sunscreen protection, may compromise vitamin D cutaneous synthesis [98]. In addition, glucocorticoid use and the proposed existence of anti-vitamin D antibodies may contribute to vitamin D deficiency [99,100,101].

The first studies about vitamin D and SLE date back to 1979, when decreased levels of the active vitamin were reported in serum of prednisone-treated juvenile SLE patients [102]. As observed in other autoimmune diseases, vitamin D deficiency has been widely described in SLE patients and has been linked to disease activity and potentially to the underlying pathogenesis [103]. Vitamin D levels inversely correlate with disease activity scores, along with anti-dsDNA and anti-complement component 1q (C1q) antibody levels [104]. However, whether vitamin D deficiency is a cause or a consequence of SLE is still not fully elucidated [105,106].

As reported in Table 2, several studies have been performed to evaluate the effects of vitamin D and other VDR agonists in SLE, initially focusing on animal models. Moreover, many observational and ex vivo studies on samples derived from SLE patients have been reported in the literature (Table 3), establishing the groundwork for subsequent interventional studies.

Back in 1992, Lemire J.M. et al. reported a decreased degree of proteinuria, autoantibody titers, and skin lesions in calcitriol-treated MRL/1 mice, an animal model of SLE, compared to untreated mice [107]. Despite these findings, no significant effects of calcitriol on proteinuria and on autoantibody production have been reported [107]. Additionally, the impact of vitamin D on renal pathology remains controversial, with conflicting results across studies. Indeed, a study on a New Zealand Black × New Zealand White F1 (NZB × W F1) murine model described aggravation of kidney histology in female mice treated with cholecalciferol, the inactive form of vitamin D [108]. More recently, Freitas et al., in pristane-induced lupus (PIL) mice, did not evidence effects of calcitriol on proteinuria, IgM and IgG deposition, and kidney histology compared to unsupplemented mice, suggesting the incapacity of vitamin D to alleviate renal injury [109]. Interestingly, beneficial effects have also been reported about the use of VDR agonists in LN. In 2022, Li X. et al. described attenuation of this condition in calcitriol-treated MRL/lpr mice. In particular, by suppressing the NF-κB and MAPK pathways, calcitriol mitigates inflammatory responses and ameliorates renal injury in lupus-prone mice [110].

Paricalcitol (9-nor-1,25-diydroxyvitamin D) treatment of Murphy Roths Large/lymphoproliferation (MRL/lpr) mice improves pathological renal alterations and decreases proteinuria and anti-dsDNA antibody levels. VDR activation can reduce anti-dsDNA antibody-induced apoptosis through the inhibition of NF-κB/NLRP3 inflammasome activation, thereby ameliorating renal pathology [111].

VDR activation and effectiveness are essential for eliciting responses to VDR agonists as potential treatments for SLE and LN, highlighting the importance of assessing its expression in relevant tissues. For instance, renal tubular epithelial cells derived from patients affected by active LN display decreased VDR expression, which inversely correlates with injury severity [113]. Moreover, VDR polymorphisms, which could impair its activation, have been associated with nephritic disorders and photosensitivity [114].

Another potential mechanism by which vitamin D may protect against renal damage is through the regulation of autophagy, which contributes to SLE pathogenesis by promoting survival of self-reactive B cells [118]. Indeed, it has been reported that vitamin D can reduce autophagy of LN-patient-derived IgG in human podocytes [115]; however, this study was performed on a small cohort, and these findings are not corroborated elsewhere.

Given the contribution of activated T cells, B cells, monocytes, and NK cells to SLE pathogenesis, the immunomodulatory properties of vitamin D may ameliorate disease course. In Act1-knockout mouse, cholecalciferol restriction (0 IU/kg, low) increased memory B cells compared to mice supplemented with high-dose cholecalciferol (10 IU/kg) for 9 weeks. Moreover, the low-cholecalciferol group showed elevated IgG levels, anti-dsDNA antibodies, and IgG deposition in the glomeruli [112]. Considering the excessive autoantibody production by B cells under low-vitamin D conditions, in vitro studies on PBMCs of SLE patients have shown that calcitriol, the active form of vitamin D, and other synthetic analogues can reduce cellular proliferation, immunoglobulin production, and the frequency of both T and B cells [116]. Moreover, reduced vitamin D levels in SLE patients have been related to enhanced B cell activation, suggesting a potential role of vitamin D deficiency in B cell hyperactivation and autoantibody production [117]. Due to the potential effects of vitamin D in SLE pathogenesis, several clinical trials have also been proposed for this condition, as reported in Table 4.

A study on a cohort of 20 female patients supplemented with cholecalciferol at a dose of 100,000 IU/week for 4 weeks followed by 100,000 IU/month for 6 months (NCT01413230) showed a significant reduction in the frequency of class-switched memory B cells after 2 months and of memory B cells at 6 months. Moreover, the authors found an increased percentage of both resting and activated memory Tregs at 2 and 6 months, along with a decrease in Th1 cells, CD8^+^ T cells, and Th17 cells, compared to the placebo group [119]. Another study in a Portuguese cohort of 24 patients reported decreased levels of Th17 cells and increased levels of Tregs after 6 months of supplementation [123].

In a randomized placebo-controlled trial (NCT01892748), 40 female juvenile SLE patients were supplemented with either 50,000 IU/week of cholecalciferol, the inactive form of vitamin D, or placebo for 6 months. At the conclusion of the study, the group receiving cholecalciferol showed an improved Systemic Lupus Erythematosus Disease Activity Index (SLEDAI), better global fatigue score, and reduced positivity to anti-dsDNA antibodies [120]. Given the reported reduction in bone density and strength in juvenile SLE patients, the same cohort was also evaluated for bone microarchitecture. The results revealed a significant increase in trabecular number, while bone mineral density remained unaffected [121].

In a randomized double-blind placebo-controlled clinical trial of 48 SLE patients (NCT00710021), cholecalciferol supplementation did not significantly decrease IFN signature, defined by the expression levels of IFN-induced genes related to disease activity [122]. However, the supplemented dose, the reduced number of patients and the limited duration of the study may not have been sufficient to provide conclusive results; therefore, further studies regarding the supplementation of vitamin D in SLE should be performed. Moreover, since sex hormones influence disease pathogenesis and treatment response [124], their effects should be taken into consideration when designing interventional studies.

### 5.4. Type 1 Diabetes Mellitus

T1D is an autoimmune disorder in which immune-mediated destruction of pancreatic β cells leads to the loss of endogenous insulin production, thereby necessitating replacement therapy. In particular, macrophages, CD8^+^ T, Th1, and Th17 cell infiltration in pancreatic islets, as well as Tregs dysfunction, contribute to disease pathogenesis [125]. Many experimental studies (Table 5) and clinical interventional studies (Table 6) have been performed to investigate the effects of VDR agonists in T1D.

Most pre-clinical studies investigating vitamin D in T1D have been conducted on non-obese diabetic (NOD) mice (Table 5). In this model, early-life vitamin D deficiency has been linked to an earlier onset and increased severity of T1D [126]. Interestingly, supplementation of vitamin D to NOD mice improves glucose tolerance. It has been proposed that supplementation downregulates CatG expression, a proteolytic enzyme involved in the modulation of the inflammatory response and glucose homeostasis, thereby inhibiting T cell activation and improving β cell function, as indicated by increased serum levels of C-peptide [127]. Studies on NOD mice supplemented with 800 IU/day vitamin D show that this treatment effectively delays T1D onset and reduces disease incidence. Moreover, increased frequency of Tregs and IL-10-secreting CD4^+^ T cells has been reported [128], highlighting the anti-inflammatory properties of vitamin D and its contribution to the modulation of immune-mediated diseases such as T1D. Based on pre-clinical results in mice, studies regarding the use of vitamin D as an adjuvant immunomodulatory therapy in T1D patients have also been performed, as reported in Table 6. Overall, vitamin D supplementation appears to enhance β cell function [129,132]; however, several studies fail to show meaningful changes in C-peptide levels in treated versus untreated individuals [130]. Moreover, supplementation seems to increase Tregs frequency [132] as well as their suppressive function [133]. Finally, in juvenile patients, vitamin D supplementation seems to decrease insulin requirements [134]. Taken together, these findings suggest a potential role of vitamin D as an adjuvant treatment in T1D.

## 6. Translational Gap Between Pre-Clinical Models and Human Autoimmune Diseases

Even though pre-clinical models have provided promising evidence regarding the immunomodulatory role of vitamin D, these findings do not translate into consistent clinical benefits in human autoimmune diseases. Indeed, studies report modest or no effects of vitamin D supplementation in autoimmune diseases; therefore, the clinical relevance of this molecule in humans remains inconsistent. The discrepancy between human and murine models highlights the limitations of translating pre-clinical studies to humans. Among the reasons for this discrepancy, species-specific differences between human and murine immune systems as well as differences in vitamin D signaling play a major role. For instance, in human VDRE sequences are located in the promoter of the IL-1β gene, while they are absent in rodents; therefore, IL-1β is not regulated by vitamin D in mice. This highlights fundamental differences in the regulation of immune-related genes in mice and humans [135]. Moreover, the distinct composition and regulation of the VDR gene in mice and humans suggest that the same stimulus may lead to different outcomes in the two species [136]. Additionally, genetic variability, including polymorphisms affecting VDR or vitamin D-metabolizing enzymes in humans, as well as interindividual biological and environmental factors, may increase the difficulties in translating animal studies in humans. Moreover, differences in dosage and timing of supplementation may explain the different findings reported in humans and mice. For instance, to reach sufficient blood levels, mice require a daily intake of 1000 IU/kg of vitamin D [137], corresponding to unrealistically high and potentially dangerous doses in humans, for whom daily intakes of approximately 200–600 IU/day are generally recommended [138]. Furthermore, it is essential to carefully control vitamin D dosage in humans to avoid the hypercalcemic side effects, which are less pronounced or more easily tolerated in murine models [139].

Taken together, these considerations highlight the importance of cautiously interpreting pre-clinical results, acknowledging species-specific mechanisms, and designing clinical studies accounting for the human immune system’s complexity and biological heterogeneity.

## 7. Conclusions

Evidence supports the role for vitamin D as a modulator of immune responses, with known effects on T cell polarization and inflammatory signaling pathways. However, the translation of these mechanistic insights into clinically meaningful benefits in autoimmune diseases remains limited. At present, results are controversial, depicting the potential immunomodulatory role of vitamin D in in vitro and in murine studies, without reaching consistent results in most clinical trials. Differences between animal models and humans should be taken into consideration while analyzing the effects of a particular compound. In humans, low vitamin D levels have been widely associated with disease activity and severity across multiple autoimmune diseases. However, interventional trials on vitamin D supplementation have given inconsistent results, considering clinical endpoints. Importantly, mechanistic evidence in pre-clinical models relies on the use of different forms of vitamin D or other VDR agonists, while clinical studies frequently rely on cholecalciferol supplementation at different dosages and schedules.

These considerations highlight the limitations of the current literature. First, as previously stated, the term “vitamin D” is frequently used imprecisely, encompassing biologically distinct compounds, which are not always specified and may have a different mechanism of action or bioavailability. Second, observational studies on vitamin D deficiency may not account for the potential confounding induced by reduced sunlight exposure, latitude, chronic inflammation, or medications taken by study participants. Moreover, clinical trials are highly heterogeneous regarding supplementation dose, duration, baseline vitamin D status, outcome measures, and statistical power, thus limiting comparability across studies and reducing the ability to detect clinically meaningful effects. Finally, improvement in immunological or molecular markers in pre-clinical models does not reliably predict the long-term clinical outcomes in the context of complex diseases, underscoring the gap between experimental outcomes and disease modifications. Taken together, current evidence does not support the benefits of vitamin D supplementation as a disease-modifying agent. Rather, vitamin D may be considered to correct deficiency and potentially modulate disease susceptibility or progression, particularly in early or pre-clinical stages.

Based on all the abovementioned considerations, future studies should aim to standardize supplementation strategies and trial designs accounting for disease heterogeneity, vitamin D metabolism, genetic background, and immune phenotype.

## Figures and Tables

**Table 1:** Summary of the clinical studies regarding the effects of cholecalciferol supplementation in patients with MS. The most relevant methodological aspects of each study are summarized in the footnotes.

Trial Name	Trial Design	Study Population	Treatment	Primary Endpoint	Findings
VIDAMS (NCT01490502) [72]	Randomized, phase 3, double-blind, multicenter, controlled trial.	172 relapsing- remitting MS (RRMS) patients.	600 IU/day (low dose) or 5000 IU/day (high dose) cholecalciferol as an add-on of glatiramer acetate daily.	Clinical relapse at 96 weeks ^1^.	No difference in relapse rate was found among patients receiving either low-dose or high-dose supplementation.
EVIDIMS (NCT01440062) [73,74]	Multicenter randomized, double-blind, phase 2a trial.	53 patients with RRMS or clinically isolated syndrome (CIS).	20,400 IU or 400 IU cholecalciferol every other day for 18 months as an add-on of IFN β-1b.	Number of new T2-weighted (T2w) hyperintense lesions on brain Magnetic Resonance Imaging (MRI) at month 18.	No significant differences in the number of T2w lesions were detected between high- or low-dose supplementation groups.
D-Lay-MS (NCT01817166) [75]	Randomized phase 3 placebo- controlled clinical trial.	303 patients with CIS suggesting MS or RRMS.	100,000 IU cholecalciferol (*n* = 156) or placebo (*n* = 147) every two weeks for 24 months.	Measure of disease activity over 24 months of follow-up ^2^.	Cholecalciferol reduces disease activity in CIS and early RRMS.
CHOLINE (NCT01198132) [76]	Phase II, multicenter, randomized, double-blind, placebo- controlled parallel-group trial.	90 RRMS patients.	7143 IU/day of cholecalciferol or placebo for 96 weeks.	Reduction in annualized relapse rate (ARR) ^3^.	Cholecalciferol supplementation significantly reduces ARR.
SOLAR (NCT01285401) [77]	Phase II, multicenter, double-blind, randomized, placebo- controlled trial.	186 patients with RRMS.	Placebo or 6670 IU/day cholecalciferol for 4 weeks followed by 14,007 IU/day up to week 48 as an add-on to IFN β-1a.	Proportion of patients with No Evidence of Disease Activity-3 (NEDA-3), Expanded Disability Status Scale (EDSS) progression, or Combined Unique Active (CUA) lesions at week 48 ^4^.	Cholecalciferol supplementation to IFN β-1a does not provide an additional effect on NEDA-3. High dose of cholecalciferol significantly reduces the number of CUA lesions, but no significant differences were found in the proportion of relapse-free patients or EDSS progression at 48 weeks.

^1^ Clinical relapse was defined as new or worsening symptoms lasting at least 24 h, accompanied by worsening in Expanded Disability Status Scale (EDSS) score or in functional system scale. The cumulative probability of clinical relapse was assessed by analyzing the time until clinical relapse using Cox’s proportional hazards model. ^2^ Disease activity was defined as occurrence of relapse or MRI activity, characterized by new and/or contrast-enhancing lesions. The Kaplan–Meier method was used to evaluate the time between treatment start and disease activity, while the log-rank test was used to compare groups. ^3^ ARR was defined as the number of relapses occurring during treatment divided by the total exposure time in years. Poisson’s regression adjusted for baseline ARR, sex, age, and EDSS was used to compare the two groups. ^4^ NEDA-3 was defined as no relapses. Logistic regression was used to analyze NEDA-3 status using EDSS score. Generalized linear models were used to analyze the cumulative CUA lesion count.

**Table 2:** Summary of experimental studies regarding the effects of VDR agonists, including cholecalciferol, calcitriol, and other derivatives, in murine models of SLE. The most relevant methodological aspects of each study are summarized in the footnotes.

Authors and Year	Study Design	Endpoint	Findings
Lemire J.M. et al., 1992 [107]	5 Murphy Roths Large/ lymphoproliferation (MRL/lpr) female mice injected with 0.1 μg calcitriol every other day for 4 weeks and then 0.15 μg for 18 weeks; 5 MRL/lpr mice in the control group received placebo.	Determination of the effects of calcitriol on proteinuria and antinuclear antibody production ^1^.	Calcitriol treatment reduces proteinuria degree and autoantibody titer.
Vaisberg M.W. et al., 2000 [108]	22 females (F) and 20 males (M) New Zealand Black × New Zealand White F1 (F1 NZB × W) mice injected with 10 μg (6 F, 5 M) or 3 μg (5 F, 5 M) cholecalciferol or placebo (11 F, 10 M) once a week for 7 months.	Evaluation of the effects of cholecalciferol treatment on kidney histology ^2^.	Cholecalciferol worsens renal histology in female mice compared to control. No significant differences were observed among males.
Freitas E.C. et al., 2019 [109]	28 female Bagg’s Albino (BALB)/c mice randomized as control group (*n* = 8), pristane- induced lupus (PIL, *n* = 10) group, or PIL injected with 2 μg/kg/day of calcitriol every two days for 6 months (*n* = 10).	Detection of immunoglobulin (Ig)G and IgM in renal tissue and histopathological evaluation. Determination of the effects of calcitriol supplementation on pristane-induced SLE-associated arthritis ^3^.	Calcitriol does not reduce renal injury or antibody deposition. However, it reduces synovial inflammation and arthritis development.
Li X. et al., 2022 [110]	48 MRL/lpr female mice injected with 4 μg/kg calcitriol twice a week for 3 weeks or placebo for 3 weeks.	Evaluation of calcitriol’s effects on kidney histology, C1q, C3, IgG and IgM deposition, NF-κB and MAPK levels, and urine protein concentration ^4^.	Calcitriol treatment ameliorates renal damage and decreases proteinuria, as well as IgM, IgG, C1q and C3 deposition. Calcitriol downregulates NF-κB and MAPK signaling, reducing inflammation and ameliorating LN.
Huang J. et al., 2021 [111]	Female MRL/lpr mice injected with 300 ng/kg paricalcitol 5 times a week for 8 weeks. Black 6 (C57BL/6) mice were used as control group and injected with placebo. Mouse Renal Tubular Epithelial Cells (mRTECs) were used to define the molecular mechanisms of paricalcitol.	Evaluation of the effects of paricalcitol on LN and molecular pathways involved ^5^.	Paricalcitol reduces proteinuria and anti-dsDNA antibodies and alleviates LN. Paricalcitol reverses anti-dsDNA antibody-induced apoptosis through the modulation of NFκB/NLRP3/caspase-1/ IL-1β/IL-18 axis in mRTECs.
Yamamoto E. et al., 2020 [112]	15 *Act1*−/− mice were fed with 0 IU/kg (low), 2 IU/kg (normal), or 10 IU/kg (high) of cholecalciferol for 9 weeks.	Effects of cholecalciferol on the development of SLE-like characteristics ^6^.	Cholecalciferol restriction promotes memory B cell development and production of autoantibodies and immunoglobulins.

^1^ Proteinuria and antibodies were measured using Enzyme-Linked Immunosorbent Assay (ELISA). ^2^ Kidney histology was assessed with Hematoxylin–Eosin (HE) and periodic acid-Shiff stains. ^3^ Kidney histology was assessed by HE stains, and IgG and IgM were detected by immunofluorescence. Arthritis was assessed by HE staining of hind paws. ^4^ Kidney histology was assessed by HE and Masson’s trichrome stains. C1q, C3, IgG, and IgM deposition were determined through immunohistochemistry (IHC). NF-κB and MAPK were detected using Western blot (WB). Proteinuria was measured with a commercial assay. ^5^ Proteinuria was assessed using a bicinchoninic acid (BCA) protein assay kit (Thermo Scientific, Waltham, MA, USA). NFκB, NLRP3, caspase-1, IL-1β, and IL-18 were detected using quantitative polymerase chain reaction (qPCR), Western blot (WB), ELISA, and IHC, ^6^ Ig were measured using ELISA. Memory B cells were identified using flow cytometry.

**Table 3:** Summary of observational and ex vivo studies regarding the role of VDR agonists, VDR expression and SNPs, and calcidiol circulating levels in SLE pathogenesis and clinical aspects. The most relevant methodological aspects of each study are summarized in the footnotes.

Authors and Year	Study Design	Methods	Endpoint	Findings
Sun J. et al., 2019 [113]	Observational	Kidney biopsies from 20 patients with LN and 5 controls ^1^.	Evaluation of VDR expression in relation to renal histology, activity scores, and proteinuria.	Renal tissue from LN patients exhibited lower VDR expression compared to controls. VDR expression was inversely correlated with both the activity index and the severity of renal injury.
De Azevêdo Silva, et al., 2013 [114]	Observational	DNA extracted using salting out from whole blood of 158 SLE patients ^2^.	Assessment of the relationship between VDR polymorphisms and risk of SLE development.	No association between VDR SNPs with SLE susceptibility has been found. VDR polymorphisms have been associated with cutaneous and immunological alterations, arthritis, anti-dsDNA antibodies, nephritic disorders, and photosensitivity.
Yu Q. et al., 2019 [115]	Ex vivo	Immortalized human podocytes (HPCs) stimulated with IgG isolated from LN patients in presence or absence of 100 nM calcitriol ^3^.	Determination of the effects of calcitriol treatment on podocyte injury.	IgG from LN patients induces podocyte injury in HPCs, which is alleviated by treatment with calcitriol.
	Observational	Renal biopsies and serum samples from 25 LN patients and 7 controls were used to determine autophagy and calcidiol levels ^4^.	Evaluation of the relationship between circulating calcidiol levels and number of autophagosomes in renal biopsies.	Autophagy is activated in renal tissue of LN patients, and it is correlated with calcidiol levels.
Linker-Israeli M. et al., 2001 [116]	Ex vivo	PBMCs isolated from 65 female SLE patients and matched healthy controls ^5^.	Evaluation of the effects of calcitriol and other synthetic analogues on cell phenotype, proliferation, and IgG production.	Calcitriol and synthetic analogues reduce proliferation and IgG production and induce B cell apoptosis.
Ritterhouse L. et al., 2011 [117]	Observational	Serum samples and PBMCs of 32 female SLE patients and matched controls ^6^.	Evaluation of the relationship between calcidiol levels, B cell hyperactivity, and autoantibody production.	Low levels of calcidiol are related with increased B cell activation.

^1^ Histological analysis was performed using HE staining, while VDR expression was detected using IHC. ^2^ VDR polymorphisms were analyzed using polymerase chain reaction (PCR) with fluorogenic allele-specific probes. ^3^ IgGs were isolated from serum using a ProteinIso Protein G Resin kit (TransGen Biotech, Beijing, China). Podocyte injury induced by autophagy was evaluated using WB for autophagy-associated proteins. ^4^ Autophagy was assessed by counting the number of autophagosomes through transmission electron microscopy (TEM) of renal biopsies, while calcidiol levels were detected in serum by an electrochemiluminescence immunoassay on serum samples. ^5^ PBMCs were isolated using Ficoll–Hypaque density gradients. Cellular subpopulations and apoptosis were analyzed using flow cytometry. PBMCs were cultured, and the supernatant was collected and analyzed for IgG presence through ELISA. ^6^ Plasma calcidiol levels were measured through an enzyme immunoassay, while autoantibodies were analyzed using an indirect immunofluorescent assay. PBMCs were isolated with Ficoll–Hypaque, and B cell activation was assessed using flow cytometry.

**Table 4:** Summary of the clinical studies regarding the effects of cholecalciferol supplementation on disease activity and immunological features in SLE patients. The most relevant methodological aspects of each study are summarized in the footnotes.

Trial Name	Trial Design	Study Population	Treatment	Primary Endpoint	Findings
VITALUP (NCT01413230) [119]	Open-label single-arm prospective clinical trial.	20 female SLE patients with hypovitaminosis D. No controls have been enrolled.	100,000 IU/week for 4 weeks followed by 100,000 IU/month cholecalciferol for 6 months. Patients were evaluated at baseline, month 2, and month 6 after supplementation.	Immunological profile of B and T cells and gene expression profile of PBMCs ^1^.	Treatment reduces memory B cells and anti-dsDNA antibodies as well as Th1 and Th17 cells.
Cholecalciferol Supplementation on Disease Activity, Fatigue and Bone Mass on Juvenile SLE (NCT01892748) [120,121]	Randomized placebo- Controlled trial.	40 female juvenile SLE patients. No controls have been enrolled.	Oral cholecalciferol at 50,000 IU/week or placebo for 6 months.	Effects of cholecalciferol supplementation on disease activity, fatigue, and bone mass ^2^.	Treatment significantly improves disease activity scores at 6 months compared to baseline, reduces fatigue at 6 months, and increases bone trabecular number at 6 months.
Vitamin D_3_ in SLE (NCT00710021) [122]	Multicenter, randomized, double-blind, placebo- controlled phase 2 study.	48 SLE patients with stable disease. No controls have been enrolled.	2000 IU (low dose), or 4000 IU (high dose) of oral cholecalciferol or placebo daily for 12 weeks.	Effects on expression of IFN-inducible genes ^3^.	No significant reduction in IFN signature has been reported in the supplemented groups.

^1^ PBMC subsets were characterized on whole blood using flow cytometry. Anti-dsDNA antibodies were detected using ELISA. Variables were compared at baseline, at month 2, and at month 6 after the start of vitamin D supplementation. ^2^ Subjects were evaluated at baseline, at 3 months, and at 6 months. Disease activity was assessed using the Systemic Lupus Erythematosus Disease Activity Index (SLEDAI) and the European Consensus Lupus Activity Measurement (ECLAM). Fatigue was assessed by a questionnaire adapted from the Fatigue Severity Scale. Bone mass and trabecular number were evaluated at the tibial site using a 3-Dimensional High-Resolution Peripheral Quantitative Computed Tomography (3D HR-pQCT) device. ^3^ Expression of IFN-inducible genes at week 12 was assessed using TaqMan Reverse Transcription PCR (RT-PCR) on whole blood.

**Table 5:** Summary of experimental studies on murine models of T1D about the effects of vitamin D on disease onset, immunological features, and β cell function. The most relevant methodological aspects of each study are summarized in the footnotes.

Author and Year	Population and Intervention	Endpoint	Findings
Giulietti et al., 2004 [126]	68 non-obese diabetic (NOD) mice fed with vitamin D-depleted diet vs. 69 control NOD mice fed with 2200 IU/kg/day vitamin D-supplemented diet for 100 days. From 100 days of age, mice were fed with supplemented diet until 250 days of age.	Investigation of the effects of vitamin D deficiency on T1D onset ^1^.	Vitamin D deficiency anticipates diabetes onset and increases severity. Increased CD4^+^ abd CD8^+^ cells and decreased Tregs infiltration have been reported in the thymus of female mice.
Lai X. et al., 2022 [127]	NOD mice were intraperitoneally injected with adenovirus carrying Cathepsin G (CatG) or Short-Hairpin RNA against CatG (sh-CatG) twice a week for 8 weeks. Mice were fed with 2200 IU/kg/day vitamin D-supplemented diet for 28 days. Control NOD mice were fed with diet containing the dietary requirements of vitamin D.	Investigation of the effects of vitamin D supplementation on CatG expression, CD4^+^ cell activation, and β cell function ^2^.	Vitamin D supplementation downregulates CatG expression, decreases CD4^+^ cell activation, improves β cell function, and inhibits apoptosis.
Martens P. et al., 2022 [128]	93 female NOD mice were fed with either cholecalciferol-sufficient diet (control diet) or diet supplemented with 400 IU/day or 800 IU/day cholecalciferol until 25 weeks of age.	Evaluation of the effects of different doses of cholecalciferol supplementation on disease onset and immunological profile ^3^.	800 IU/day supplementation decreases T1D development and increases FOXP3^+^ Tregs and IL-10-secreting CD4^+^ T cell frequency.

^1^ T1D onset was assessed measuring the levels of glucose in urine and blood samples two times a week. Thymuses from mice at 100 days of age were removed and dispensed into single-cell suspensions, and cell subtypes were analyzed using flow cytometry. ^2^ Pancreatic tissue was embedded in paraffin, while mononuclear cells were isolated using Percoll gradient. PBMCs were isolated using the Ficoll–Hypaque technique. CD4^+^ T cells were sorted from pancreatic mononuclear cells using magnetic beads coated with anti-CD4 antibody. CD4^+^ T cell activation was analyzed using flow cytometry. Proteins were isolated from pancreatic tissues and cells using radioimmunoprecipitation assay lysis buffer with protease inhibitor. β cell apoptosis was assessed by measuring the protein levels of B cell lymphoma 2 (BCL-2), BCL-2-Associated X protein (BAX), cleaved caspase-3, and cleaved caspase-9 using the WB technique. β cell function was assessed measuring serum C-peptide levels using ELISA. ^3^ T1D onset was evaluated measuring urine and blood glucose concentrations. Spleen, pancreatic lymph nodes, and mesenteric lymph nodes single-cell suspensions were isolated by mechanical disruption of tissues at 8 and 25 weeks of age. Cell subsets were analyzed using flow cytometry.

**Table 6:** Summary of the interventional studies on T1D patients regarding the effects of vitamin D supplementation on immunological landscape, β cell function, glycated hemoglobin, and calcidiol levels. The most relevant methodological aspects of each study are summarized in the footnotes.

Study Design	Study Population and Treatment	Primary Endpoint	Findings
Prospective, multicenter, open-label, randomized. (ADVENT, NCT02407899) [129].	301 T1D patients randomized to (I) metformin with or without insulin (conventional therapy, CT, *n* = 99), (II) CT plus saxagliptin (*n* = 100), or (III) CT plus saxagliptin plus 2000 IU cholecalciferol/day (*n* = 102) for 24 months.	Evaluation of β cell function measured by C-peptide levels ^1^.	Improvement in β cell function loss between supplemented and conventional therapy groups, especially in patients with high Glutamic Acid Decarboxylase Autoantibody (GADA) levels.
Multicenter, randomized, double-blind, placebo- controlled trial [130].	109 T1D patients received three intralymphatic injections with 4 μg Diamyd^®^ on days 30, 60, and 90 and 2000 IU/day oral vitamin D for 4 months (*n* = 57) or placebo in place of each treatment (*n* = 52). Last study visit was performed at month 15.	Changes in serum C-peptide levels over the 2 h period after a mixed-meal tolerance test (MMTT) ^2^.	No differences in C-peptide levels between baseline and 15-month visit were reported.
Controlled clinical trial [131].	133 young T1D patients randomized to cholecalciferol at 2000 IU/day (*n* = 103) or placebo (*n* = 30) for 12 weeks.	Measurement of glycated hemoglobin (HbA1c) ^3^.	Minimal effects on glycemic control have been reported.
Randomized, double-blind, placebo- controlled study [132].	35 new-onset T1D patients randomized to 2000 IU cholecalciferol (*n* = 17) or placebo (*n* = 18) for 18 months.	Evaluation of cytokines, chemokines, HbA1c, and C-peptide levels as well as Tregs frequency ^4^.	C-peptide levels increased at 12 months from diagnosis, and the decline was reduced at 18 months compared to controls. Increase in Tregs frequency was reported after 12 months. No differences in HbA1c levels have been identified.
Prospective, randomized, double-blind, placebo- controlled trial (NCT01390480) [133].	30 juvenile T1D patients supplemented weekly with cholecalciferol (corresponding to 70 IU/kg/day) or placebo for 12 months.	Evaluation of changes in frequency and function of Tregs ^5^.	Tregs frequency did not differ between baseline and month 12. Suppressive function of Tregs increased at month 12.
Cross-sectional study [134].	141 juvenile T1D patients supplemented with 1000 IU/day cholecalciferol.	Evaluation of the effects of vitamin D supplementation on glycemic status ^6^.	Supplementation significantly increases serum calcidiol levels and reduces HbA1c at final visit. Calcidiol levels inversely correlate with insulin requirement.

^1^ Absolute change in fasting C-peptide levels from baseline to month 24 was compared between the two treatment groups and the conventional therapy group. A mixed model for repeated measures used to analyze absolute change was adjusted for age, sex, time, treatment-by-time interaction, baseline C-peptide, baseline C-peptide-by-time interaction, baseline vitamin D concentration, insulin or metformin dosage, and diabetic ketoacidosis (DKA). Stratification of patients according to GADA levels at baseline was used to assess treatment effects on β cell function. ^2^ C-peptide levels were measured using a dual-sided chemiluminescence immunoassay. Changes in stimulated C-peptide levels were assessed at 15 months versus baseline between the treatment and placebo groups. ^3^ HbA1c levels were measured using high-performance liquid chromatography (HPLC). A paired t-test was used to evaluate variables before and after treatment. Models were adjusted by age, sex, and time of T1D onset. ^4^ HbA1c levels in whole blood were measured using HPLC. C-peptide levels were measured using an immunofluorometric assay. Cytokines and chemokines from serum samples were measured using inflammatory cytokine and chemokine bead array kits (Becton Dickinson, Franklin Lakes, NJ, USA). Tregs in PBMCs were analyzed according to the manufacturer’s instructions (eBioscience, San Diego, CA, USA). Repeated-measures analysis of variance was used to compare C-peptide and HbA1c levels in the treatment and placebo groups. Pearson’s and multivariate regression analyses were used to correlate variables. ^5^ PBMCs were isolated from whole blood using Histopaque density gradient. The absolute number of leukocytes was measured using a cell counter, while Tregs analysis was performed using flow cytometry. Tregs and T effector cells were isolated using a cell sorter. To assess the suppressive function of Tregs, T effector cells were cultivated in the presence or absence of autologous Tregs and in the presence of irradiated PBMCs. T effector cells were stimulated with CD3/CD28-coated microbeads, and proliferation was assessed by analyzing ^3^H-thymidine uptake. ^6^ Subjects were analyzed at disease onset (T0, *n* = 64), at 12–24 months before last visit (T1, *n* = 124), and at last visit (T2). The average time of supplementation was 17.0 ± 9.5 months at T1 and 17.7 ± 8.1 months at T2. HbA1c levels were measured by HPLC. Calcidiol levels were measured by a direct competitive chemiluminescent immunoassay. Variation between T1 and T2 was evaluated by a paired t test for repeated measures. Pearson’s correlation analysis was used to correlate calcidiol levels to clinical parameters.

## Data Availability

No new data were created or analyzed in this study. Data sharing is not applicable to this article.

## Abbreviations

The following abbreviations are used in this manuscript:

1,24,25(OH)_3_D_3_	1,24,25-Trihydroxycholecalciferol
24,25(OH)_2_D_3_	24,25-Dihydroxycholecalciferol
3D HR-pQCT	3-Dimensional High-Resolution Peripheral Quantitative Computed Tomography
Ab	Antibody
ACPA	Anti-Citrullinated Protein Antibodies
anti-C1q	Anti-Complement Component 1q
anti-dsDNA	Anti-Double-Strand DNA
AP-1	Activator Protein 1
ARR	Annualized Relapse Rate
BACH	BTB Domain And CNC Homolog 1
BALB	Bagg’s Albino
BAX	BCL2 Associated X, Apoptosis Regulator (Bax)
BBB	Blood–Brain Barrier
BCA	Bicinchoninic Acid
BCL-2	B Cell Lymphoma 2
C57BL/6	Black 6
CatG	Cathepsin G
CD	Cluster Differentiation
CIS	Clinically Isolated Syndrome
CNS	Central Nervous System
CRP	C-Reactive Protein
CT	Conventional Therapy
CTLA-4	Cytotoxic T-Lymphocyte Antigen 4
CUA	Combined Unique Active
CYP	Cytochrome P450
CYP27B1	1α-Hydroxylase
DCs	Dendritic Cells
DKA	Diabetic Ketoacidosis
EAE	Experimental Autoimmune Encephalomyelitis
ECLAM	European Consensus Lupus Activity Measurement
EDSS	Expanded Disability Status Scale
ERK	Extracellular-Signal-Regulated Kinases
F	Female
FOXP3	Forkhead Box P3
FSMC	Fatigue Scale for Motor and Cognitive Functions
GADA	Glutamic Acid Decarboxylase Autoantibodies
GATA-3	GATA Binding Protein 3
Gfi1	Growth Factor Independent 1 Transcriptional Repressor
GM-CSF	Granulocyte–Macrophage Colony-Stimulating Factor
HbA1c	Glycated Hemoglobin
HE	Hematoxylin–Eosin
HPCs	Immortalized Human Podocytes
HPLC	High-Performance Liquid Chromatography
IBD	Inflammatory Bowel Disease
IFN	Interferon
Ig	Immunoglobulin
IHC	Immunohistochemistry
IKKβ	Inhibitor Of Nuclear Factor Kappa B Kinase Subunit Beta
IL	Interleukin
IRF4	Interferon Regulatory Factor 4
IU	International Units
IκBα	Nuclear Factor Of Kappa Light Polypeptide Gene Enhancer in B cells Inhibitor Alpha
JAK	Janus Kinase
JNK	c-Jun N-Terminal Kinase
LILRB4	Leukocyte Immunoglobulin-Like Receptor Subfamily B member 4
LN	Lupus Nephritis
LPS	Lipopolysaccharide
M	Male
MAPK	Mitogen-Activated Protein Kinase
mDCs	Myeloid-Derived DCs
MHC	Major Histocompatibility Complex
miR	Micro-RNA
MMTT	Mixed-Meal Tolerance Test
MRI	Magnetic Resonance Imaging
MRL/lpr	Murphy Roths Large/lymphoproliferation
mRTECs	Mouse Renal Tubular Epithelial Cells
MS	Multiple Sclerosis
MTX	Methotrexate
NEDA-3	No Evidence of Disease Activity-3
NF-κB	Nuclear Factor Kappa-Light-Chain-Enhancer of Activated B Cells
NFAT	Nuclear Factor of Activated T cells
NLRP3	NLR Family Pyrin Domain Containing 3
NOD	Non-Obese Diabetic
NZB × W F1	New Zealand Black x New Zealand White F1
PAMPs	Pathogen-Associated Molecular Patterns
PBMCs	Peripheral Blood Mononuclear Cells
PCR	Polymerase Chain Reaction
PD-L1	Programmed Death-Ligand 1
pDCs	Plasmacytoid DCs
PIL	Pristane-induced Lupus
PTH	Parathyroid Hormone
qPCR	Quantitative Polymerase Chain Reaction
RA	Rheumatoid Arthritis
RF	Rheumatoid Factor
RRMS	Relapsing–Remitting Multiple Sclerosis
RT-PCR	Reverse Transcription Polymerase Chain Reaction
RXR	Retinoid-X-Receptor
sh-CatG	Short-Hairpin RNA against CatG
SLE	Systemic Lupus Erythematosus
SLEDAI	Systemic Lupus Erythematosus Disease Activity Index
SNPs	Single Nucleotide Polymorphisms
SOCS	Suppressor of Cytokine Signaling
STAT	Signal Transducer and Activator of Transcription
T1D	Type 1 Diabetes Mellitus
T2w	T2-weighted
tDCs	Tolerogenic DCs
TEM	Transmission Electron Microscopy
TGF	Transforming Growth Factor
Th	T Helper Cells
TLR	Toll-Like Receptors
TNF	Tumor Necrosis Factor
Tregs	Regulatory T Cells
UV	Ultraviolet
VDBP	Vitamin D Binding Protein
VDR	Vitamin D Receptor
VDR−/− hTNFtg	VDR Knockout Human Tumor Necrosis Factor α Transgenic
VDREs	Vitamin D Response Elements
WB	Western Blot

## References

[1] Papagni R., Pellegrino C., Di Gennaro F., Patti G., Ricciardi A., Novara R., Cotugno S., Musso M., Guido G., Ronga L. (2022). Impact of Vitamin D in Prophylaxis and Treatment in Tuberculosis Patients. Int. J. Mol. Sci..

[2] Chun R.F., Liu P.T., Modlin R.L., Adams J.S., Hewison M. (2014). Impact of Vitamin D on Immune Function: Lessons Learned from Genome-Wide Analysis. Front. Physiol..

[3] Sun L., Arbesman J., Piliang M. (2021). Vitamin D. Autoimmunity and Immune-Related Adverse Events of Immune Checkpoint Inhibitors. Arch. Dermatol. Res..

[4] Formisano E., Proietti E., Borgarelli C., Pisciotta L. (2023). Psoriasis and Vitamin D: A Systematic Review and Meta-Analysis. Nutrients.

[5] Brożyna A.A., Slominski R.M., Nedoszytko B., Zmijewski M.A., Slominski A.T. (2022). Vitamin D Signaling in Psoriasis: Pathogenesis and Therapy. Int. J. Mol. Sci..

[6] Vernia F., Valvano M., Longo S., Cesaro N., Viscido A., Latella G. (2022). Vitamin D in Inflammatory Bowel Diseases. Mechanisms of Action and Therapeutic Implications. Nutrients.

[7] Gorini F., Tonacci A. (2024). Vitamin D: An Essential Nutrient in the Dual Relationship between Autoimmune Thyroid Diseases and Celiac Disease-A Comprehensive Review. Nutrients.

[8] Infantino C., Francavilla R., Vella A., Cenni S., Principi N., Strisciuglio C., Esposito S. (2022). Role of Vitamin D in Celiac Disease and Inflammatory Bowel Diseases. Nutrients.

[9] Durá-Travé T., Gallinas-Victoriano F. (2024). Autoimmune Thyroiditis and Vitamin D. Int. J. Mol. Sci..

[10] Holick M.F. (2006). Resurrection of Vitamin D Deficiency and Rickets. J. Clin. Investig..

[11] Christakos S., Dhawan P., Verstuyf A., Verlinden L., Carmeliet G. (2015). Vitamin D: Metabolism, Molecular Mechanism of Action, and Pleiotropic Effects. Physiol. Rev..

[12] Holick M.F., Binkley N.C., Bischoff-Ferrari H.A., Gordon C.M., Hanley D.A., Heaney R.P., Murad M.H., Weaver C.M. (2011). Evaluation, Treatment, and Prevention of Vitamin D Deficiency: An Endocrine Society Clinical Practice Guideline. J. Clin. Endocrinol. Metab..

[13] Holick M.F. (2007). Vitamin D Deficiency. N. Engl. J. Med..

[14] Jones G., Prosser D.E., Kaufmann M. (2014). Cytochrome P450-Mediated Metabolism of Vitamin D. J. Lipid Res..

[15] Dusso A.S., Brown A.J., Slatopolsky E., Vitamin E.S., Slatopolsky E. (2005). Vitamin D. Am. J. Physiol. Renal Physiol..

[16] Khammissa R.A.G., Fourie J., Motswaledi M.H., Ballyram R., Lemmer J., Feller L. (2018). The Biological Activities of Vitamin D and Its Receptor in Relation to Calcium and Bone Homeostasis, Cancer, Immune and Cardiovascular Systems, Skin Biology, and Oral Health. Biomed. Res. Int..

[17] Bikle D.D. (2014). Vitamin D Metabolism, Mechanism of Action, and Clinical Applications. Chem. Biol..

[18] Takeyama K.I., Kato S. (2011). The Vitamin D3 Lalpha-Hydroxylase Gene and Its Regulation by Active Vitamin D3. Biosci. Biotechnol. Biochem..

[19] Khundmiri S.J., Murray R.D., Lederer E. (2016). PTH and Vitamin D. Compr. Physiol..

[20] Hii C.S., Ferrante A. (2016). The Non-Genomic Actions of Vitamin D. Nutrients.

[21] Rosen C.J., Adams J.S., Bikle D.D., Black D.M., Demay M.B., Manson J.A.E., Murad M.H., Kovacs C.S. (2012). The Nonskeletal Effects of Vitamin D: An Endocrine Society Scientific Statement. Endocr. Rev..

[22] Marshall J.S., Warrington R., Watson W., Kim H.L. (2018). An Introduction to Immunology and Immunopathology. Allergy Asthma Clin. Immunol..

[23] Liu P.T., Stenger S., Li H., Wenzel L., Tan B.H., Krutzik S.R., Ochoa M.T., Schauber J., Wu K., Meinken C. (2006). Toll-like Receptor Triggering of a Vitamin D-Mediated Human Antimicrobial Response. Science.

[24] Adams J.S., Hewison M. (2008). Unexpected Actions of Vitamin D: New Perspectives on the Regulation of Innate and Adaptive Immunity. Nat. Clin. Pract. Endocrinol. Metab..

[25] Charoenngam N., Holick M.F. (2020). Immunologic Effects of Vitamin d on Human Health and Disease. Nutrients.

[26] Aranow C. (2011). Vitamin D and the Immune System. J. Investig. Med..

[27] Hewison M. (2010). Vitamin D and the Intracrinology of Innate Immunity. Mol. Cell Endocrinol..

[28] Stoffels K., Overbergh L., Giulietti A., Verlinden L., Bouillon R., Mathieu C. (2006). Immune Regulation of 25-Hydroxyvitamin-D3-1α-Hydroxylase in Human Monocytes. J. Bone Miner. Res..

[29] Chen Y., Liu W., Sun T., Huang Y., Wang Y., Deb D.K., Yoon D., Kong J., Thadhani R., Li Y.C. (2013). 1,25-Dihydroxyvitamin D Promotes Negative Feedback Regulation of TLR Signaling via Targeting MicroRNA-155–SOCS1 in Macrophages. J. Immunol..

[30] Cohen-Lahav M., Shany S., Tobvin D., Chaimovitz C., Douvdevani A. (2006). Vitamin D Decreases NFκB Activity by Increasing IκBα Levels. Nephrol. Dial. Transplant..

[31] Zhang Y., Leung D.Y.M., Richers B.N., Liu Y., Remigio L.K., Riches D.W., Goleva E. (2012). Vitamin D Inhibits Monocyte/Macrophage Proinflammatory Cytokine Production by Targeting MAPK Phosphatase-1. J. Immunol..

[32] Adorini L., Penna G. (2009). Dendritic Cell Tolerogenicity: A Key Mechanism in Immunomodulation by Vitamin D Receptor Agonists. Hum. Immunol..

[33] Suuring M., Moreau A. (2021). Regulatory Macrophages and Tolerogenic Dendritic Cells in Myeloid Regulatory Cell-Based Therapies. Int. J. Mol. Sci..

[34] Hewison M. (2012). Vitamin D and Immune Function: An Overview. Proc. Nutr. Soc..

[35] Vanherwegen A.S., Gysemans C., Mathieu C. (2017). Vitamin D Endocrinology on the Cross-Road between Immunity and Metabolism. Mol. Cell Endocrinol..

[36] Penna G., Adorini L. (2000). 1α,25-Dihydroxyvitamin D3 Inhibits Differentiation, Maturation, Activation, and Survival of Dendritic Cells Leading to Impaired Alloreactive T Cell Activation. J. Immunol..

[37] Wu D., Lewis E.D., Pae M., Meydani S.N. (2019). Nutritional Modulation of Immune Function: Analysis of Evidence, Mechanisms, and Clinical Relevance. Front. Immunol..

[38] Rolf L., Muris A.H., Hupperts R., Damoiseaux J. (2014). Vitamin D Effects on B Cell Function in Autoimmunity. Ann. New York Acad. Sci..

[39] Heine G., Niesner U., Chang H.D., Steinmeyer A., Zügel U., Zuberbier T., Radbruch A., Worm M. (2008). 1,25-Dihydroxyvitamin D3 Promotes IL-10 Production in Human B Cells. Eur. J. Immunol..

[40] Ghaseminejad-Raeini A., Ghaderi A., Sharafi A., Nematollahi-Sani B., Moossavi M., Derakhshani A., Sarab G.A. (2023). Immunomodulatory Actions of Vitamin D in Various Immune-Related Disorders: A Comprehensive Review. Front. Immunol..

[41] Artusa P., White J.H. (2025). Vitamin D and Its Analogs in Immune System Regulation. Pharmacol. Rev..

[42] Cantorna M.T., Snyder L., Lin Y.D., Yang L. (2015). Vitamin D and 1,25(OH)_2_D Regulation of T Cells. Nutrients.

[43] Jeffery L.E., Burke F., Mura M., Zheng Y., Qureshi O.S., Hewison M., Walker L.S.K., Lammas D.A., Raza K., Sansom D.M. (2009). 1,25-Dihydroxyvitamin D3 and IL-2 Combine to Inhibit T Cell Production of Inflammatory Cytokines and Promote Development of Regulatory T Cells Expressing CTLA-4 and FoxP3. J. Immunol..

[44] Van Etten E., Mathieu C. (2005). Immunoregulation by 1,25-Dihydroxyvitamin D3: Basic Concepts. J. Steroid Biochem. Mol. Biol..

[45] Lauretani F., Salvi M., Zucchini I., Testa C., Cattabiani C., Arisi A., Maggio M. (2023). Relationship between Vitamin D and Immunity in Older People with COVID-19. Int. J. Environ. Res. Public. Health.

[46] Jirapongsananuruk O., Melamed I., Leung D.Y.M. (2000). Additive Immunosuppressive Effects of 1,25-Dihydroxyvitamin D3 and Corticosteroids on T(H)1, but Not T(H)2, Responses. J. Allergy Clin. Immunol..

[47] Biswas B., Chattopadhyay S., Hazra S., Goswami R. (2024). Calcitriol Impairs the Secretion of IL-4 and IL-13 in Th2 Cells via Modulating the VDR-Gata3-Gfi1 Axis. J. Immunol..

[48] Staeva-Vieira T.P., Freedman L.P. (2002). 1,25-Dihydroxyvitamin D3 Inhibits IFN-γ and IL-4 Levels During In Vitro Polarization of Primary Murine CD4+ T Cells. J. Immunol..

[49] Chauss D., Freiwald T., McGregor R., Yan B., Wang L., Nova-Lamperti E., Kumar D., Zhang Z., Teague H., West E.E. (2022). Autocrine Vitamin D Signaling Switches off Pro-Inflammatory Programs of TH1 Cells. Nat. Immunol..

[50] Wimalawansa S.J. (2023). Infections and Autoimmunity—The Immune System and Vitamin D: A Systematic Review. Nutrients.

[51] Athanassiou L., Kostoglou-Athanassiou I., Koutsilieris M., Shoenfeld Y. (2023). Vitamin D and Autoimmune Rheumatic Diseases. Biomolecules.

[52] Vitamin D—Health Professional Fact Sheet. https://ods.od.nih.gov/factsheets/VitaminD-HealthProfessional/.

[53] Hahn J., Cook N.R., Alexander E.K., Friedman S., Walter J., Bubes V., Kotler G., Lee I.M., Manson J.A.E., Costenbader K.H. (2022). Vitamin D and Marine Omega 3 Fatty Acid Supplementation and Incident Autoimmune Disease: VITAL Randomized Controlled Trial. BMJ.

[54] Jolliffe D.A., Walton R.T., Griffiths C.J., Martineau A.R. (2016). Single Nucleotide Polymorphisms in the Vitamin D Pathway Associating with Circulating Concentrations of Vitamin D Metabolites and Non-Skeletal Health Outcomes: Review of Genetic Association Studies. J. Steroid Biochem. Mol. Biol..

[55] Agliardi C., Guerini F.R., Bolognesi E., Zanzottera M., Clerici M. (2023). VDR Gene Single Nucleotide Polymorphisms and Autoimmunity: A Narrative Review. Biology.

[56] Melake A., Mengstie M.A. (2025). Vitamin D Deficiency and VDR TaqI Polymorphism on Diabetic Nephropathy Risk among Type 2 Diabetes Patients. Front. Endocrinol..

[57] Kafentzi T., Tsounis E.P., Tourkochristou E., Avramopoulou E., Aggeletopoulou I., Geramoutsos G., Sotiropoulos C., Pastras P., Thomopoulos K., Theocharis G. (2025). Genetic Polymorphisms (ApaI, FokI, BsmI, and TaqI) of the Vitamin D Receptor (VDR) Influence the Natural History and Phenotype of Crohn’s Disease. Int. J. Mol. Sci..

[58] Imani D., Razi B., Motallebnezhad M., Rezaei R. (2019). Association between Vitamin D Receptor (VDR) Polymorphisms and the Risk of Multiple Sclerosis (MS): An Updated Meta-Analysis. BMC Neurol..

[59] Mohammadi A., Azarnezhad A., Khanbabaei H., Izadpanah E., Abdollahzadeh R., Barreto G.E., Sahebkar A. (2020). Vitamin D Receptor Genetic Polymorphisms and the Risk of Multiple Sclerosis: A Systematic Review and Meta-Analysis. Steroids.

[60] Pakosiński M., Żyła M., Kamieniak A., Kluz N., Gil-Kulik P. (2025). Vitamin D Receptor Polymorphisms and Immunological Effects of Vitamin D in Hashimoto’s Thyroiditis. Int. J. Mol. Sci..

[61] Latini A., De Benedittis G., Perricone C., Colafrancesco S., Conigliaro P., Ceccarelli F., Chimenti M.S., Novelli L., Priori R., Conti F. (2021). VDR Polymorphisms in Autoimmune Connective Tissue Diseases: Focus on Italian Population. J. Immunol. Res..

[62] Ram V.S., Sharma M., Anbhule A.P., Jaison A., Info A. (2025). Exploring Genetic Variability in VDR FokI and BsmI Polymorphisms and Their Association with Rheumatoid Arthritis. EJIFCC.

[63] Song G.G., Bae S.C., Lee Y.H. (2016). Vitamin D Receptor FokI, BsmI, and TaqI Polymorphisms and Susceptibility to Rheumatoid Arthritis: A Meta-Analysis. Z. Rheumatol..

[64] Zhou T.B., Jiang Z.P., Lin Z.J., Su N. (2015). Association of Vitamin D Receptor Gene Polymorphism with the Risk of Systemic Lupus Erythematosus. J. Recept. Signal Transduct. Res..

[65] Liu R., Du S., Zhao L., Jain S., Sahay K., Rizvanov A., Lezhnyova V., Khaibullin T., Martynova E., Khaiboullina S. (2022). Autoreactive Lymphocytes in Multiple Sclerosis: Pathogenesis and Treatment Target. Front. Immunol..

[66] Balasooriya N.N., Elliott T.M., Neale R.E., Vasquez P., Comans T., Gordon L.G. (2024). The Association between Vitamin D Deficiency and Multiple Sclerosis: An Updated Systematic Review and Meta-Analysis. Mult. Scler. Relat. Disord..

[67] Faridar A., Eskandari G., Sahraian M.A., Minagar A., Azimi A. (2012). Vitamin D and Multiple Sclerosis: A Critical Review and Recommendations on Treatment. Acta Neurol. Belg..

[68] Sailike B., Onzhanova Z., Akbay B., Tokay T., Molnár F. (2024). Vitamin D in Central Nervous System: Implications for Neurological Disorders. Int. J. Mol. Sci..

[69] Kočovská E., Gaughran F., Krivoy A., Meier U.C. (2017). Vitamin-D Deficiency as a Potential Environmental Risk Factor in Multiple Sclerosis, Schizophrenia, and Autism. Front. Psychiatry.

[70] Shirazi H.A., Rasouli J., Ciric B., Wei D., Rostami A., Zhang G.X. (2017). 1,25-Dihydroxyvitamin D3 Suppressed Experimental Autoimmune Encephalomyelitis through Both Immunomodulation and Oligodendrocyte Maturation. Exp. Mol. Pathol..

[71] de Oliveira L.R.C., Mimura L.A.N., Fraga-Silva T.F.d.C., Ishikawa L.L.W., Fernandes A.A.H., Zorzella-Pezavento S.F.G., Sartori A. (2020). Calcitriol Prevents Neuroinflammation and Reduces Blood-Brain Barrier Disruption and Local Macrophage/Microglia Activation. Front. Pharmacol..

[72] Cassard S.D., Fitzgerald K.C., Qian P., Emrich S.A., Azevedo C.J., Goodman A.D., Sugar E.A., Pelletier D., Waubant E., Mowry E.M. (2023). High-Dose Vitamin D3 Supplementation in Relapsing-Remitting Multiple Sclerosis: A Randomised Clinical Trial. EClinicalMedicine.

[73] Dörr J., Bäcker-Koduah P., Wernecke K.D., Becker E., Hoffmann F., Faiss J., Brockmeier B., Hoffmann O., Anvari K., Wuerfel J. (2020). High-Dose Vitamin D Supplementation in Multiple Sclerosis—Results from the Randomized EVIDIMS (Efficacy of Vitamin D Supplementation in Multiple Sclerosis) Trial. Mult. Scler. J. Exp. Transl. Clin..

[74] Gomez-Figueroa E., Moreno-Bernardino C., Bäcker-Koduah P., Dörr J., Rubarth K., Konietschke F., Bellmann-Strobl J., Ruprecht K., Oertel F.C., Paul F. (2025). Neuroprotective Role of High Dose Vitamin D Supplementation in Multiple Sclerosis: Sub-Analysis of the EVIDIMS Trial. Mult. Scler. Relat. Disord..

[75] Thouvenot E., Laplaud D., Lebrun-Frenay C., Derache N., Le Page E., Maillart E., Froment-Tilikete C., Castelnovo G., Casez O., Coustans M. (2025). High-Dose Vitamin D in Clinically Isolated Syndrome of Multiple Sclerosis The D-Lay MS Randomized Clinical Trial. JAMA.

[76] Camu W., Lehert P., Pierrot-Deseilligny C., Hautecoeur P., Besserve A., Deleglise A.S.J., Payet M., Thouvenot E., Souberbielle J.C. (2019). Cholecalciferol in Relapsing-Remitting MS: A Randomized Clinical Trial (CHOLINE). Neurol. Neuroimmunol. Neuroinflamm..

[77] Hupperts R., Smolders J., Vieth R., Holmøy T., Marhardt K., Schluep M., Killestein J., Barkhof F., Beelke M., Grimaldi L.M.E. (2019). Randomized Trial of Daily High-Dose Vitamin D3 in Patients with RRMS Receiving Subcutaneous Interferon β-1a. Neurology.

[78] Smolders J., Hupperts R., Barkhof F., Grimaldi L.M.E., Holmoy T., Killestein J., Rieckmann P., Schluep M., Vieth R., Hostalek U. (2011). Efficacy of Vitamin D3 as Add-on Therapy in Patients with Relapsing-Remitting Multiple Sclerosis Receiving Subcutaneous Interferon Beta-1a: A Phase II, Multicenter, Double-Blind, Randomized, Placebo-Controlled Trial. J. Neurol. Sci..

[79] Muris A.H., Smolders J., Rolf L., Thewissen M., Hupperts R., Damoiseaux J. (2016). Immune Regulatory Effects of High Dose Vitamin D3 Supplementation in a Randomized Controlled Trial in Relapsing Remitting Multiple Sclerosis Patients Receiving IFNβ; the SOLARIUM Study. J. Neuroimmunol..

[80] Sokolova M.V., Schett G., Steffen U. (2022). Autoantibodies in Rheumatoid Arthritis: Historical Background and Novel Findings. Clin. Rev. Allergy Immunol..

[81] Kinne R.W., Bräuer R., Stuhlmüller B., Palombo-Kinne E., Burmester G.R. (2000). Macrophages in Rheumatoid Arthritis. Arthritis Res..

[82] Ishikawa L.L.W., Colavite P.M., Fraga-Silva T.F.d.C., Mimura L.A.N., França T.G.D., Zorzella-Pezavento S.F.G., Chiuso-Minicucci F., Marcolino L.D., Penitenti M., Ikoma M.R.V. (2017). Vitamin D Deficiency and Rheumatoid Arthritis. Clin. Rev. Allergy Immunol..

[83] Neve A., Corrado A., Cantatore F.P. (2014). Immunomodulatory Effects of Vitamin D in Peripheral Blood Monocyte-Derived Macrophages from Patients with Rheumatoid Arthritis. Clin. Exp. Med..

[84] Zwerina K., Baum W., Axmann R., Heiland G.R., Distler J.H., Smolen J., Hayer S., Zwerina J., Schett G. (2011). Vitamin D Receptor Regulates TNF-Mediated Arthritis. Ann. Rheum. Dis..

[85] Srivastava S., Rasool M. (2024). Genetics, Epigenetics and Autoimmunity Constitute a Bermuda Triangle for the Pathogenesis of Rheumatoid Arthritis. Life Sci..

[86] Jang S., Kwon E.J., Lee J.J. (2022). Rheumatoid Arthritis: Pathogenic Roles of Diverse Immune Cells. Int. J. Mol. Sci..

[87] Harrison S.R., Li D., Jeffery L.E., Raza K., Hewison M. (2020). Vitamin D, Autoimmune Disease and Rheumatoid Arthritis. Calcif. Tissue Int..

[88] Li N., Wei W., Yin F., Chen M., Ma T.R., Wu Q., Zhou J.R., Zheng S.-G., Han J. (2015). The Abnormal Expression of CCR4 and CCR6 on Tregs in Rheumatoid Arthritis. Int. J. Clin. Exp. Med..

[89] Jeffery L.E., Raza K., Hewison M. (2016). Vitamin D in Rheumatoid Arthritis—Towards Clinical Application. Nat. Rev. Rheumatol..

[90] Wen H.-Y., Luo J., Li X.-F., Wei D.-D., Liu Y. (2019). 1,25-Dihydroxyvitamin D 3 Modulates T Cell Differentiation and Impacts on the Production of Cytokines from Chinese Han Patients with Early Rheumatoid Arthritis. Immunol. Res..

[91] El-Banna H.S., Gado S.E. (2020). Vitamin D: Does It Help Tregs in Active Rheumatoid Arthritis Patients. Expert. Rev. Clin. Immunol..

[92] Verma S., Chaturvedi V., Ganguly N.K., Mittal S.A. (2021). Vitamin D Deficiency: Concern for Rheumatoid Arthritis and COVID-19?. Mol. Cell Biochem..

[93] Liu Y., Wen H. (2018). Impact of Vitamin D Deficiency on Clinical Parameters in Treatment-Naïve Rheumatoid Arthritis Patients. Z. Rheumatol..

[94] Lin J., Liu J., Davies M.L., Chen W. (2016). Serum Vitamin D Level and Rheumatoid Arthritis Disease Activity: Review and Meta-Analysis. PLoS ONE.

[95] Sainaghi P.P., Bellan M., Antonini G., Bellomo G., Pirisi M. (2011). Unsuppressed Parathyroid Hormone in Patients with Autoimmune/Inflammatory Rheumatic Diseases: Implications for Vitamin D Supplementation. Rheumatology.

[96] Sainaghi P.P., Bellan M., Nerviani A., Sola D., Molinari R., Cerutti C., Pirisi M. (2013). Superiority of a High Loading Dose of Cholecalciferol to Correct Hypovitaminosis D in Patients with Inflammatory/Autoimmune Rheumatic Diseases. J. Rheumatol..

[97] Ameer M.A., Chaudhry H., Mushtaq J., Khan O.S., Babar M., Hashim T., Zeb S., Tariq M.A., Patlolla S.R., Ali J. (2022). An Overview of Systemic Lupus Erythematosus (SLE) Pathogenesis, Classification, and Management. Cureus.

[98] Hile G.A., Coit P., Xu B., Victory A.M., Gharaee-Kermani M., Estadt S.N., Maz M.P., Martens J.W.S., Wasikowski R., Dobry C. (2023). Regulation of Photosensitivity by the Hippo Pathway in Lupus Skin. Arthritis Rheumatol..

[99] de Carvalho J.F., Skare T.L., Martinez A.T.A., Shoenfeld Y. (2025). Anti-Vitamin D Antibodies. Autoimmun. Rev..

[100] Carvalho J.F., Blank M., Kiss E., Tarr T., Amital H., Shoenfeld Y. (2007). Anti-Vitamin D, Vitamin D in SLE. Ann. N. Y. Acad. Sci..

[101] Yaniv G., Twig G., Shor D.B.A., Furer A., Sherer Y., Mozes O., Komisar O., Slonimsky E., Klang E., Lotan E. (2015). A Volcanic Explosion of Autoantibodies in Systemic Lupus Erythematosus: A Diversity of 180 Different Antibodies Found in SLE Patients. Autoimmun. Rev..

[102] O’Regan S., Chesney R.W., Hamstra A., Eisman J.A., O’Gorman A.M., Deluca H.F. (1979). Reduced Serum 1,25-(OH)_2_ Vitamin D3 Levels in Prednisone-Treated Adolescents with Systemic Lupus Erythematosus. Acta Paediatr..

[103] Breslin L.C., Magee P.J., Wallace J.M.W., McSorley E.M. (2011). An Evaluation of Vitamin D Status in Individuals with Systemic Lupus Erythematosus. Proc. Nutr. Soc..

[104] Mok C.C., Birmingham D.J., Ho L.Y., Hebert L.A., Song H., Rovin B.H. (2012). Vitamin D Deficiency as Marker for Disease Activity and Damage in Systemic Lupus Erythematosus: A Comparison with Anti-DsDNA and Anti-C1q. Lupus.

[105] Ding Y., Yang S., Fan S., Tang Y., Teng Y., Tao X., Lu W. (2022). Is Vitamin D Deficiency the Cause or the Effect of Systemic Lupus Erythematosus: Evidence from Bidirectional Mendelian Randomization Analysis. J. Immunol. Res..

[106] Islam M.A., Khandker S.S., Alam S.S., Kotyla P., Hassan R. (2019). Vitamin D Status in Patients with Systemic Lupus Erythematosus (SLE): A Systematic Review and Meta-Analysis. Autoimmun. Rev..

[107] Lemire J.M., Ince A., Takashima M. (1992). 1,25-Dihydroxyvitamin D3 Attenuates of Expression of Experimental Murine Lupus of MRL/1 Mice. Autoimmunity.

[108] Vaisberg M.W., Kaneno R., Franco M.F., Mendes N.F. (2000). Influence of Cholecalciferol (Vitamin D3) on the Course of Experimental Systemic Lupus Erythematosus in F1 (NZB×W) Mice. J. Clin. Lab. Anal..

[109] Correa Freitas E., Evelyn Karnopp T., de Souza Silva J.M., Cavalheiro do Espírito Santo R., da Rosa T.H., de Oliveira M.S., da Costa Gonçalves F., de Oliveira F.H., Guilherme Schaefer P., André Monticielo O. (2019). Vitamin D Supplementation Ameliorates Arthritis but Does Not Alleviates Renal Injury in Pristane-Induced Lupus Model. Autoimmunity.

[110] Li X., Liu J., Zhao Y., Xu N., Lv E., Ci C., Li X. (2022). 1,25-Dihydroxyvitamin D3 Ameliorates Lupus Nephritis through Inhibiting the NF-ΚB and MAPK Signalling Pathways in MRL/Lpr Mice. BMC Nephrol..

[111] Huang J., An Q., Ju B., Zhang J., Fan P., He L., Wang L. (2021). Role of Vitamin D/VDR Nuclear Translocation in down-Regulation of NF-ΚB/NLRP3/Caspase-1 Axis in Lupus Nephritis. Int. Immunopharmacol..

[112] Yamamoto E.A., Nguyen J.K., Liu J., Keller E., Campbell N., Zhang C.J., Smith H.R., Li X., Jørgensen T.N. (2020). Low Levels of Vitamin D Promote Memory B Cells in Lupus. Nutrients.

[113] Sun J., Zhang S., Liu J.S., Gui M., Zhang H. (2019). Expression of Vitamin D Receptor in Renal Tissue of Lupus Nephritis and Its Association with Renal Injury Activity. Lupus.

[114] De Azevêdo Silva J., Fernandes K.M., Pancotto J.A.T., Fragoso T.S., Donadi E.A., Crovella S., Sandrin-Garcia P. (2013). Vitamin D Receptor (VDR) Gene Polymorphisms and Susceptibility to Systemic Lupus Erythematosus Clinical Manifestations. Lupus.

[115] Yu Q., Qiao Y., Liu D., Liu F., Gao C., Duan J., Liang L., Di X., Yuan Y., Gao Y. (2019). Vitamin D Protects Podocytes from Autoantibodies Induced Injury in Lupus Nephritis by Reducing Aberrant Autophagy. Arthritis Res. Ther..

[116] Linker-Israeli M., Elstner E., Klinenberg J.R., Wallace D.J., Koeffler H.P. (2001). Vitamin D3 and Its Synthetic Analogs Inhibit the Spontaneous in Vitro Immunoglobulin Production by SLE-Derived PBMC. Clin. Immunol..

[117] Ritterhouse L.L., Crowe S.R., Niewold T.B., Kamen D.L., Macwana S.R., Roberts V.C., Dedeke A.B., Harley J.B., Scofield R.H., Guthridge J.M. (2011). Vitamin D Deficiency Is Associated with an Increased Autoimmune Response in Healthy Individuals and in Patients with Systemic Lupus Erythematosus. Ann. Rheum. Dis..

[118] Raza I.G.A., Clarke A.J. (2021). B Cell Metabolism and Autophagy in Autoimmunity. Front. Immunol..

[119] Terrier B., Derian N., Schoindre Y., Chaara W., Geri G., Zahr N., Mariampillai K., Rosenzwajg M., Carpentier W., Musset L. (2012). Restoration of Regulatory and Effector T Cell Balance and B Cell Homeostasis in Systemic Lupus Erythematosus Patients through Vitamin D Supplementation. Arthritis Res. Ther..

[120] Lima G.L., Paupitz J., Aikawa N.E., Takayama L., Bonfa E., Pereira R.M.R. (2016). Vitamin D Supplementation in Adolescents and Young Adults with Juvenile Systemic Lupus Erythematosus for Improvement in Disease Activity and Fatigue Scores: A Randomized, Double-Blind, Placebo-Controlled Trial. Arthritis Care Res..

[121] Lima G.L., Paupitz J.A., Aikawa N.E., Alvarenga J.C., Pereira R.M.R. (2018). A Randomized Double-Blind Placebo-Controlled Trial of Vitamin D Supplementation in Juvenile-Onset Systemic Lupus Erythematosus: Positive Effect on Trabecular Microarchitecture Using HR-PQCT. Osteoporos. Int..

[122] Aranow C., Kamen D.L., Dall’Era M., Massarotti E.M., MacKay M.C., Koumpouras F., Coca A., Chatham W.W., Clowse M.E.B., Criscione-Schreiber L.G. (2015). Randomized, Double-Blind, Placebo-Controlled Trial of the Effect of Vitamin D3 on the Interferon Signature in Patients with Systemic Lupus Erythematosus. Arthritis Rheumatol..

[123] Marinho A., Carvalho C., Boleixa D., Bettencourt A., Leal B., Guimarães J., Neves E., Oliveira J.C., Almeida I., Farinha F. (2017). Vitamin D Supplementation Effects on FoxP3 Expression in T Cells and FoxP3+/IL-17A Ratio and Clinical Course in Systemic Lupus Erythematosus Patients: A Study in a Portuguese Cohort. Immunol. Res..

[124] Kim J.W., Kim H.A., Suh C.H., Jung J.Y. (2022). Sex Hormones Affect the Pathogenesis and Clinical Characteristics of Systemic Lupus Erythematosus. Front Med..

[125] Zajec A., Trebušak Podkrajšek K., Tesovnik T., Šket R., Čugalj Kern B., Jenko Bizjan B., Šmigoc Schweiger D., Battelino T., Kovač J. (2022). Pathogenesis of Type 1 Diabetes: Established Facts and New Insights. Genes.

[126] Giulietti A., Gysemans C., Stoffels K., Van Etten E., Decallonne B., Overbergh L., Bouillon R., Mathieu C. (2004). Vitamin D Deficiency in Early Life Accelerates Type 1 Diabetes in Non-Obese Diabetic Mice. Diabetologia.

[127] Lai X., Liu X., Cai X., Zou F. (2022). Vitamin D Supplementation Induces CatG-Mediated CD4þ T Cell Inactivation and Restores Pancreatic b-Cell Function in Mice with Type 1 Diabetes. Am. J. Physiol. Endocrinol. Metab..

[128] Martens P.J., Centelles-Lodeiro J., Ellis D., Cook D.P., Sassi G., Verlinden L., Verstuyf A., Raes J., Mathieu C., Gysemans C. (2022). High Serum Vitamin D Concentrations, Induced via Diet, Trigger Immune and Intestinal Microbiota Alterations Leading to Type 1 Diabetes Protection in NOD Mice. Front. Immunol..

[129] Yan X., Li X., Liu B., Huang J., Xiang Y., Hu Y., Tang X., Zhang Z., Huang G., Xie Z. (2023). Combination Therapy with Saxagliptin and Vitamin D for the Preservation of β-Cell Function in Adult-Onset Type 1 Diabetes: A Multi-Center, Randomized, Controlled Trial. Signal Transduct. Target. Ther..

[130] Ludvigsson J., Sumnik Z., Pelikanova T., Chavez L.N., Lundberg E., Rica I., Martínez-Brocca M.A., de Adana M.R., Wahlberg J., Katsarou A. (2021). Intralymphatic Glutamic Acid Decarboxylase with Vitamin d Supplementation in Recent-Onset Type 1 Diabetes: A Double-Blind, Randomized, Placebo-Controlled Phase IIb Trial. Diabetes Care.

[131] Figueiredo Moreira C.F., Ferreira Peres W.A., Silva do Nascimento Braga J., Proença da Fonseca A.C., Junior M.C., Luescher J., Campos L., de Carvalho Padilha P. (2025). Effect of Vitamin D Supplementation on Glycemic Control in Children and Adolescents with Type 1 Diabetes Mellitus: Data from a Controlled Clinical Trial. Diabetes Res. Clin. Pract..

[132] Gabbay M.A.L., Sato M.N., Finazzo C., Duarte A.J.S., Dib S.A. (2012). Effect of Cholecalciferol as Adjunctive Therapy with Insulin on Protective Immunologic Profile and Decline of Residual β-Cell Function in New-Onset Type 1 Diabetes Mellitus. Arch. Pediatr. Adolesc. Med..

[133] Treiber G., Prietl B., Fröhlich-Reiterer E., Lechner E., Ribitsch A., Fritsch M., Rami-Merhar B., Steigleder-Schweiger C., Graninger W., Borkenstein M. (2015). Cholecalciferol Supplementation Improves Suppressive Capacity of Regulatory T-Cells in Young Patients with New-Onset Type 1 Diabetes Mellitus—A Randomized Clinical Trial. Clin. Immunol..

[134] Savastio S., Cadario F., Genoni G., Bellomo G., Bagnati M., Secco G., Picchi R., Giglione E., Bona G. (2016). Vitamin D Deficiency and Glycemic Status in Children and Adolescents with Type 1 Diabetes Mellitus. PLoS ONE.

[135] Dimitrov V., White J.H. (2016). Species-Specific Regulation of Innate Immunity by Vitamin D Signaling. J. Steroid Biochem. Mol. Biol..

[136] Marcinkowska E. (2020). The Vitamin D System in Humans and Mice: Similar but Not the Same. Reports.

[137] National Research Council (US) Subcommittee on Laboratory Animal Nutrition (1995). Nutrient Requirements of Laboratory Animals.

[138] World Health Organization, Food and Agriculture Organization of the United Nations (2004). Recommended intakes for vitamin D. Vitamin and Mineral Requirements in Human Nutrition.

[139] Fletcher J., Bishop E.L., Harrison S.R., Swift A., Cooper S.C., Dimeloe S.K., Raza K., Hewison M. (2022). Autoimmune Disease and Interconnections with Vitamin D. Endocr Connect..

